# Protocol for fast antibiotic resistance-based gene editing of mammalian cells with CRISPR-Cas9

**DOI:** 10.1016/j.xpro.2025.103949

**Published:** 2025-08-04

**Authors:** Petia Adarska, Eleanor Fox, Joshua Heyza, Carlo Barnaba, Jens Schmidt, Francesca Bottanelli

**Affiliations:** 1 Freie Universität Berlin, Institute of Chemistry and Biochemistry, Thielallee 63, 14195 Berlin, Germany; 2 Institute for Quantitative Health Science and Engineering, Michigan State University, East Lansing, MI, USA; 3 Department of Obstetrics, Gynecology, and Reproductive Biology, Michigan State University, East Lansing, MI, USA

**Keywords:** Cell Biology, Molecular Biology, CRISPR

## Abstract

Protein tagging with CRISPR-Cas9 enables the investigation of protein function in its native environment but is limited by low homology-directed repair (HDR) efficiency. Here, we present a protocol for fast antibiotic resistance-based gene editing with CRISPR-Cas9 (FAB-CRISPR), which streamlines N/C-terminal tagging using an antibiotic resistance cassette for rapid selection and enrichment of gene-edited cells. We describe in detail guide RNA and HDR donor plasmid cloning, transfection of editing reagents into HeLa cells, and subsequent enrichment and verification of gene-edited cells.

For complete details on the use and execution of this protocol, please refer to Wong-Dilworth et al.,^1^ Stockhammer et al.,^2^ Stockhammer et al.,^3^ Heyza et al.,^4^ and Broadbent et al.^5^

## Highlights

• Guidance on endogenous tagging with CRISPR-Cas9 in mammalian cells
• Procedure for boosting gene editing efficiency via an antibiotic resistance cassette
• Steps for design and cloning of editing reagents for cell line generation and validation

## Abstract (editor highlights)

Publisher’s note: Undertaking any experimental protocol requires adherence to local institutional guidelines for laboratory safety and ethics.

## Graphical abstract

**Figure:**
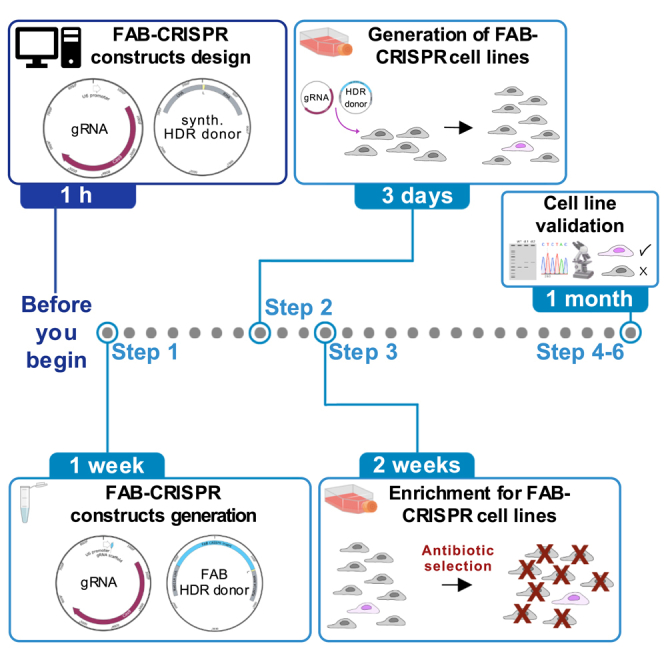

## Before you begin

**Figure 1:**
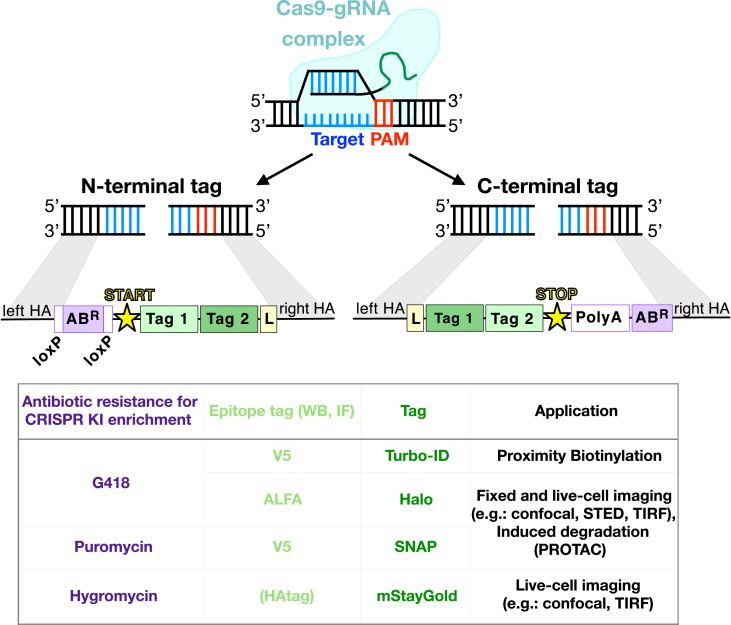
Overview of the available HDR donor plasmids with the various Tag-antibiotic resistance cassette combinations We present various FAB-CRISPR insert combinations that can be used to generate KIs for various downstream applications including proximity-based proteomics, live-cell microscopy [*e.g.,* stimulated emission depletion (STED) and total internal reflection fluorescence (TIRF)] and induced degradation using proteolysis targeting chimera (PROTAC). Monomeric StayGold (mStayGold), linker (L). Not drawn to scale.

The site-specific introduction of a DNA double-strand break (DSB) by clustered regularly interspaced short palindromic repeats (CRISPR)-Cas9 allows for targeted genomic alterations that enable protein knock-out (KO) and knock-in (KI).^6^^,^^7^^,^^8^ A major hurdle of gene editing is the low efficiency of homology-directed repair (HDR) which results in KI efficiency in the low percentage range. Here, we present a step-by-step protocol for endogenous protein tagging in human cells, which overcomes low HDR efficiency through an integrated antibiotic resistance cassette (AB^R^, for details on the antibiotic resistance cassette see Supplement Figure S1). Our workflow guides users from reagent design to cell line generation and validation. The provided FAB-CRISPR HDR donor plasmids contain pre-designed inserts for both C- and N-terminal tagging of a variety of Tag and AB^R^ combinations (Figure 1). These Tags enable a range of downstream application including live-cell microscopy (SNAP,^9^ Halo^10^) and the new highly photostable green fluorescent protein monomeric StayGold (mStayGold, https://www.fpbase.org/protein/mstaygold/)^11^ as well as proximity-based proteomics^3^^,^^12^ (Turbo-ID). Additionally, SNAP and Halo Tags have been used to acutely modulate protein function using proteolysis targeting chimera (PROTAC) substrates.^13^^,^^14^^,^^15^ The FAB-CRISPR inserts can be easily copied and pasted into a HDR donor plasmid of choice in a single cloning step. For tagging a protein of interest, we have implemented a single-step (C-terminal tagging) or a two-step (N-terminal tagging) genome editing strategy. The first step enables antibiotic selection of successfully edited cells that have integrated the Tag and an AB^R^ sequence from the HDR donor plasmid. The pipeline is highly adaptable for introducing any exogenous DNA fragment into transfection-compatible cell genomes and enables the enrichment of cells edited with non-fluorescent tags (*e.g.* Turbo-ID and short epitope tags). Antibiotic selection allows for dramatic enrichment of low efficiency editing events and is particularly beneficial for low expressed endogenous targets where the signal may be barely detectable above background when attempting Fluorescence-activated cell sorting (FACS) before enrichment. In addition, background derived from the labeling of cells with cell permeable SNAP and Halo substrates may also make FACS challenging when working with low efficiency and low abundance targets. Genes involved in a wide range of cellular processes, such as membrane trafficking,^1^^,^^2^^,^^3^^,^^15^^,^^16^ autophagy,^5^^,^^17^^,^^18^ DNA repair,^19^^,^^20^^,^^21^^,^^22^^,^^23^ and telomere maintenance^24^^,^^25^^,^^26^ have also been successfully targeted using this method. Our method has been applied to introduce many other protein Tags (*e.g*., mEOS3.2, enhanced GFP (EGFP), APEX2, and small epitope Tags) into multiple mammalian cell lines, including epithelial cell lines (U-2 OS, RPE-1, T84, HAP1, MCF7, MCF10A, Caco2), T cells (Jurkat) and mast cell lines (RBL-2H3, LUVA).^1^^,^^2^^,^^5^^,^^17^

Two components are required to generate KI cell lines. First, a 20 nucleotide long guide RNA (gRNA) is required to target the Cas9 nuclease to the genomic region of interest, leading to the introduction of a DSB, with cleavages on each DNA strand directly opposite each other. Second, HDR DNA template is necessary to promote homologous recombination of the targeted alleles. The HDR donor plasmid harbors the sequence to be knocked-in, flanked by homology arms (HAs) with homology to the genomic sequences upstream and downstream of the DSB locus. This ultimately leads to a generally error-free, site-specific insertion of the sequence of interest.^27^

In this first preparation section, we will guide you through the process of designing a gRNA and a HDR donor plasmid for the generation of KI cell lines using the FAB-CRISPR inserts.

### gRNA design for tagging the N or C terminus of a protein of interest

**Timing: 30 min**

Here, we will describe how to select a suitable gRNA sequence for KIs using the Benchling CRISPR Guide design tool.

**CRITICAL:** When selecting a gRNA, it is important to ensure accurate insertion of the donor sequence, minimize the risk of generating indels or point mutations at the insertion site, and prevent cleavage of the HDR donor plasmid to avoid compromising KI efficiency.

1. Decide at which terminus your protein of interest should be tagged to retain functionality.
2. Go to www.benchling.com and create an account.
3. Create a new project by clicking on the “+” icon (indicated by the red box) (Figure 2).

**Figure 2:**
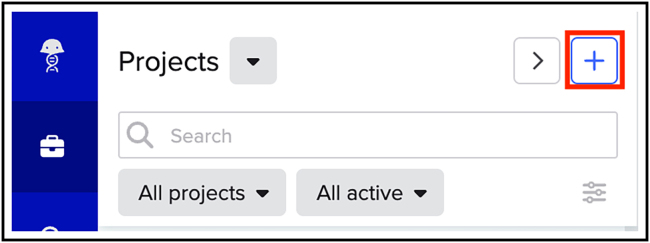
How to create a new Project in Benchling
4. To import the genomic sequence of interest, click on the “+” icon next to your newly created project and select “DNA/RNA sequence“, followed by “import DNA/RNA sequences.” Then select “import from external database“ and search for your gene of interest in the sequence field, as shown in the example searches (Figure 3). Enter “human” in the genome search field to select the GRCh38 human annotated genome. Click on the Search button.

**Figure 3:**
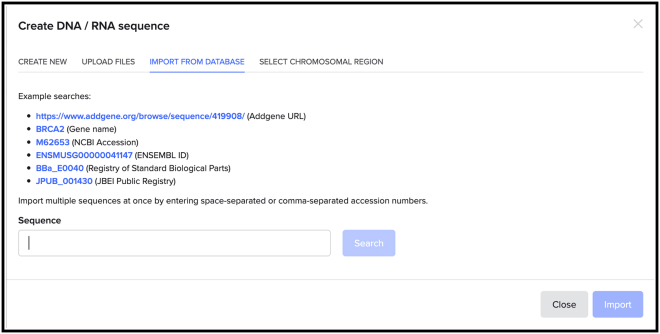
How to import the desired genomic sequence

***Note:*** This is essential to select a gRNA sequence that will target the Cas9 to induce a DSB in an appropriate site and to design the HDR donor plasmid for editing.

**Figure 4:**
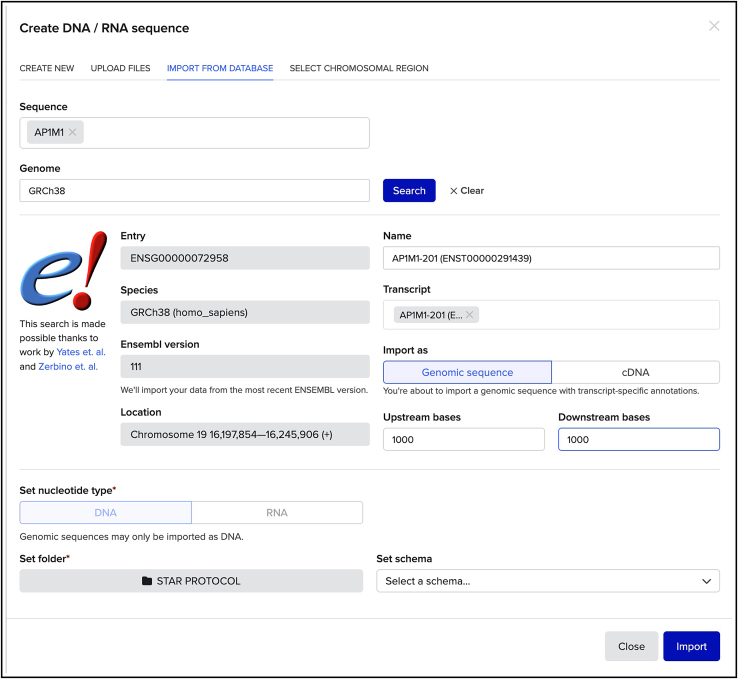
How to visualize the genomic region of interest for designing the HDR donor plasmid and gRNA The AP1M1 genomic locus is used as an example.

***Note:*** This will give you information on the location of your locus of interest, so where exactly the sequence of interest is on a specific chromosome.

***Note:*** The default transcript is typically the canonical sequence.

5. In the next window (Figure 4), select which transcript to use for further visualization on Benchling.

**CRITICAL:** Import the sequence with an extra 1000 bp upstream and downstream of the locus, which will be important later for the design of the HDR donor plasmid.

***Note:*** To obtain information about possible splicing isoforms and transcripts search for your protein of interest on www.uniprot.org. One has to be particularly careful when alternative splicing yields protein products with different N- or C-termini and whether the edited DNA may affect splicing. Another important consideration is that N- or C-terminal tagging of a protein will label all splice isoforms, regardless of differences in their untagged terminus or internal sequences.

**Figure 5:**
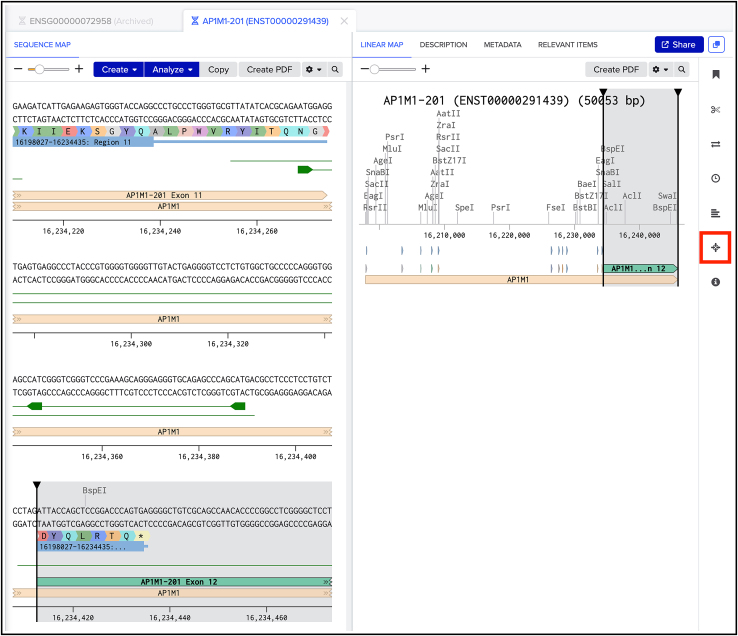
How to design a gRNA with Benchling Click the star shaped icon (highlighted by a red box) for designing gRNAs targeting the selected genomic region. Here the C-terminal region of AP1M1 is visualized as an example.

***Note:*** The START/STOP codon will be used to guide the design of the homology arms (HAs) for the HDR donor plasmid, as the Tag will be inserted into the genomic locus upstream of the START codon (N-terminal tagging) or downstream of the last aminoacidic-encoding codon (C-terminal tagging).

6. Identify the START/STOP codon of the gene of interest (Figure 5) and click on the arrows in the linear map (right panel, Figure 5) to visualize specific exons.

7. Select the site where you want to insert your Tag (at the N- or C-terminus) and highlight a sequence of +/− 50 bp around the insertion site to design the gRNA by selecting the sequence in the sequence map window (left panel, Figure 5).

***Note:*** In the example shown (Figure 5), we have selected a 100 bp sequence around the STOP codon, located on the last exon, of the AP1M1 gene locus encoding for AP1μA, a protein involved in intracellular trafficking.

**Figure 6:**
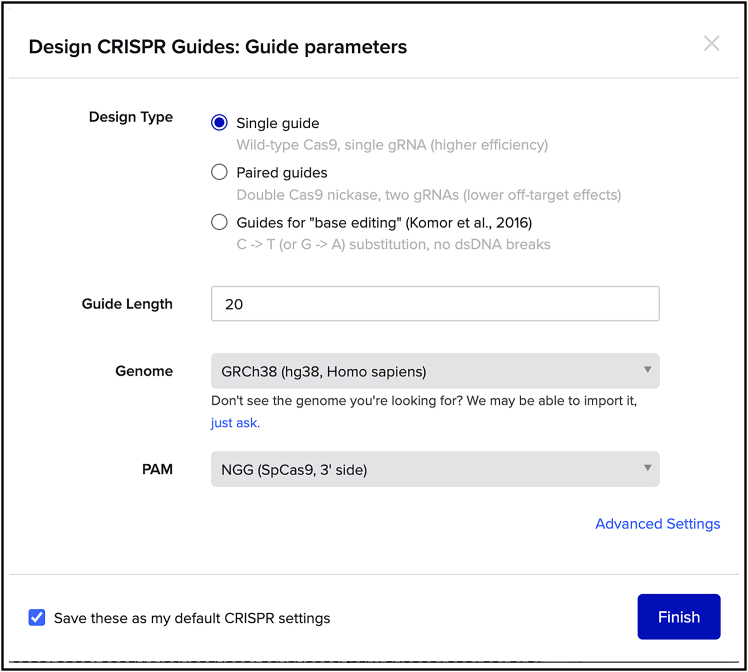
Parameters for designing a gRNA cutting in the selected genomic region when using *Streptococcus pyogenes* Cas9 (SpCas9)

***Note:*** All the visualized guides will contain a PAM sequence (NGG for SpCas9) at the 3′ end as a PAM sequence is necessary for the recognition of the target sequence by the Cas9 complex.

8. Click on the star-shaped icon on the very right of the page (CRISPR button, highlighted by a red square in Figure 5) and select “design and analyze guides”.
9. Select guide parameters as in the example shown below when using wild-type Cas9 from *Streptococcus pyogenes* Cas9 (SpCas9) (Figure 6).

10. Click on “Finish” (Figure 6) to confirm the selected parameters and generate a list of putative gRNAs target sequences in your selected region on either the sense (+) or anti-sense (−) strands.

***Note:*** When working with guide RNAs on the anti-sense (−) strand remember to design your oligonucleotides from 5′ to 3′.

**Figure 7:**
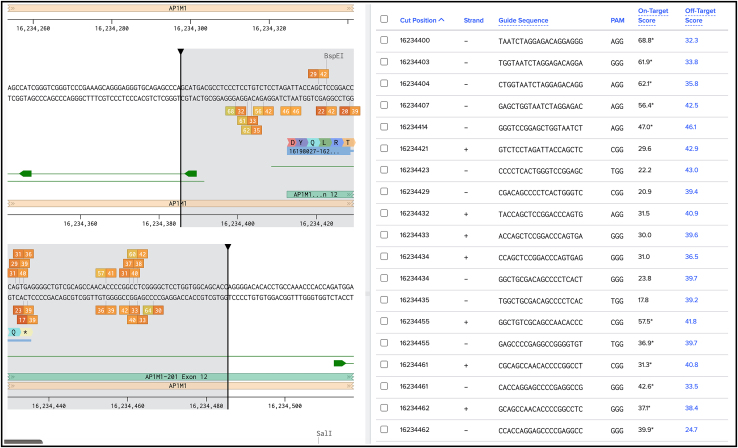
How to select a gRNA List of possible gRNAs cutting around the end of the AP1M1 coding region.

11. Click on a specific guide sequence to visualize in the sequence map window on the left and choose two guides from the list of available gRNA target sequences shown on the right (Figure 7).

**CRITICAL:** For choosing an optimal guide consider closeness to the insertion site, the highest on-target score^28^ and no off-targets.^29^

**CRITICAL:** Check that no other genes are targeted by the selected guide. The specificity of the selected guide is indicated by an off-target score (a high off-target score is better). Click on the off-target score of the specific guide you are interested in for visualizing possible off-target sequences and the probability of off-target effects.

***Note:*** For N-terminal tagging, we recommend choosing a gRNA that cuts after the START codon. The efficiency of Cas9 cutting and thus non-homologous end-joining (NHEJ) that leads to allele KO is very high, whereas HDR is a rare event. If cutting by the Cas9 occurs after the START codon, cells with one KI allele, where HDR has occurred, and one KO allele, where NHEJ has occurred, could be created. Despite this heterozygosity at the gene level, these cells may appear homozygous at the protein level (to be confirmed via Western Blot). For C-terminal tagging, it is important to choose a guide that will cut after the STOP codon. If cutting by the Cas9 occurs before the STOP codon, cells will likely bear one or more alleles (depending on the ploidity of the cell line used) with mutations and/or frameshifting indels at the very 3′ of the open reading frame (ORF). This is undesirable when wanting to work with a mixed population of cells as some cells will likely express proteins with altered C-termini.

***Note:*** If possible, choose a gRNA sequence that spans the STOP (or START) codon, meaning the complete gRNA sequence would not be included in the HDR donor plasmid, protecting it from enzymatic cleavage by the Cas9. This will also simplify HDR donor plasmid design (see next Major Step), as the introduction of a silent mutation in the region of homology to remove the PAM site is not required (Figure 8).

**Figure 9:**
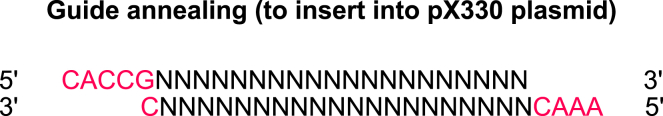
How to design oligos for gRNA cloning into the pX330 plasmid To allow cloning of the selected gRNA in pX330, the designed oligonucleotides need overhangs (indicated in red) that allow ligation of the annealed oligos into the pX330 plasmid digested with BpiI.

***Note:*** Ordering the correct oligos is critical to ensure proper annealing.

12. Order two complementary oligonucleotides (oligos) for the selected gRNA sequence, which must include the 20 bases of the gRNA and the additional overhangs for cloning into a pX330 plasmid (see Figure S2 for a plasmid map) for simultaneous expression of SpCas9 and the gRNA (Figure 9).^8^^,^^30^

**Figure 8:**
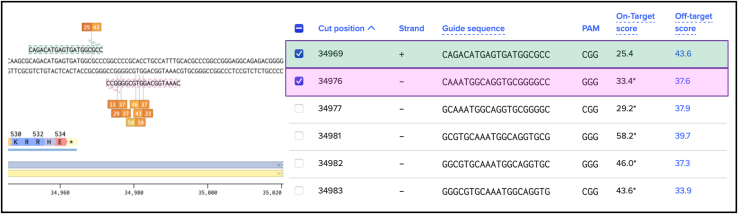
Example showing possible gRNA targeting the sequence downstream of a STOP codon The green gRNA spans over the STOP codon (on the + strand) so that no silent mutation of the PAM site in the HDR donor plasmid is needed. The magenta gRNA (on the - strand) recognizes a target sequence after the STOP codon, requiring the introduction of silent mutations in the HDR donor plasmids to prevent cutting by the Cas9. The shown example is for the selection of gRNAs targeting the locus of EHD1.

Oligonucleotide #1: CACCGNNNNNNNNNNNNNNNNNNNN.

Oligonucleotide #2: AAACNNNNNNNNNNNNNNNNNNNNC.

### Designing a homology-directed repair donor plasmid for N- or C-terminal tagging

**Timing: 30 min**

Here, we will describe how to design for each target gene a HDR donor plasmid consisting of a left homology arm (LHA), two unique restriction sites for cloning of the Tag and the AB^R^ and a right homology arm (RHA).

***Note:*** HDR donor plasmids can be synthesized using a DNA synthesis service of your choice.

**Figure 10:**
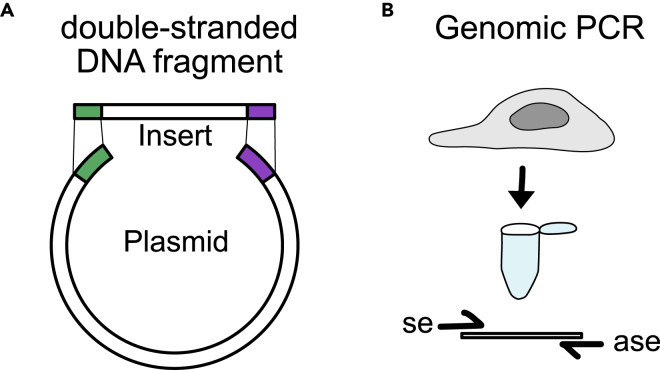
Alternative methods for obtaining homology arms (HAs) (A) HAs can be synthesized as a double-stranded DNA fragment containing overlapping sequences with the backbone plasmid of choice (indicated in green and violet) to allow cloning via Gibson assembly. (B) Alternatively, HAs can be amplified from genomic DNA via PCR for restriction-digest-based cloning. Polymerase chain reaction (PCR), se (sense), ase (anti-sense).

***Alternatives*:** Gibson assembly or standard genomic polymerase chain reaction (PCR) can be used to clone the HAs into your plasmid of choice (Figure 10). Plasmids can be assembled *in silico* in a molecular biology software such as SnapGene to generate a target sequence map.

With a single cloning step, you will then be able to copy and paste the pre-designed inserts: Tag-polyA-AB^R^ (for C-terminal fusions) or a LoxP-AB^R^-LoxP-Tag (for N-terminal fusions) from the provided FAB-CRISPR plasmids collection (described later in Major Steps “Generation of HDR donor plasmids for N/C-terminal tagging”, for an overview of the available FAB-CRISPR inserts see Figure 1).

***Note:*** For C-terminal protein tagging, the antibiotic resistance cassette (AB^R^) is placed downstream of the Tag and an exogenous polyadenylation (polyA) sequence. For N-terminal fusions, the AB^R^ is upstream of the Tag, or within the Tag coding sequence, and is flanked by LoxP sites allowing for removal of the antibiotic resistance gene by EGFP-Cre mediated recombination.^31^

***Note:*** The pre-designed FAB-CRISPR inserts can also be customized to generate various AB^R^/Tag combinations using restriction sites that are present between the AB^R^ and Tag (Figures S3A and S3B) or by exchanging parts with a custom AB^R^/Tag that is generated through PCR from a plasmid of choice (Figure S3C), for more details see Major Step “Customizing FAB-CRISPR HDR donor plasmid”.

13. Use Benchling to select 500 bp sequences downstream/upstream of the START codon (N-terminal tagging) or downstream/upstream from the STOP codon (C-terminal tagging).

***Alternatives*:** The genomic sequence of interest can be obtained from https://genome.ucsc.edu/. For this, click on “Genome Browser” and then enter the gene of interest. Afterward click on the gene name on the far-left side of the screen and in the new window click on “Genomic Sequence” under “Sequence and Links to Tools and Databases”. In the subsequent window, include the promoter, 5′ UTR, CDS exons, introns, 3′ UTR, and downstream bases and click submit for downloading the genomic sequence of interest.

14. Make sure to include two restriction sites in between the two HAs to be able to copy and paste Tags and resistance markers from the suggested FAB-CRISPR plasmids (as described later in Major Steps “Generation of HDR donor plasmids for N/C-terminal tagging”).

***Note:*** For N-terminal tagging, design the HDR donor plasmid as LHA-NheI-NNNNN-BamHI-L-RHA. For C-terminal tagging design the HDR donor plasmid as LHA-L-BamHI-NNNNN-EcoRI-RHA, where NNNNN are random bases to facilitate restriction digests and L is a glycine-serine linker of choice.

**CRITICAL:** If the suggested restriction enzymes cut within your designed HDR donor plasmid, mutagenize the recognition sequences by inserting silent point mutations (within the coding sequence). We have successfully introduced mutations in introns without altering the levels of downstream protein products. Alternatively, use different restriction sites and amplify the Tag and antibiotic resistance cassettes via PCR.

**CRITICAL:** If one of the homology arms contains the gRNA sequence, the Cas9 will cleave the HDR donor plasmid (see Figure 8). To overcome this issue, a mutation should be introduced to disrupt the PAM sequence on the HDR donor plasmid. When mutating the PAM sequence is not possible, introduce 2-3 single point mutations in the upstream 20 base sequence recognized by the Cas9. In the case of N-terminal tagging, it is important to introduce a silent mutation that will not alter the protein product. (If no silent mutations can be introduced, choose another gRNA.)

***Note:*** The HDR donor plasmid can be synthesized from any nucleic acid supplier as a DNA sequence in a high copy number plasmid (such as pUC plasmids) with the preferred bacterial antibiotic resistance.

**CRITICAL:** Before proceeding with the synthesis, verify that the *in silico* assembled HDR donor plasmid contains a START codon immediately preceding the Tag for N-terminal fusions. For C-terminal fusions, verify the presence of a STOP codon at the end of the Tag fragment.

***Note:*** In both the N- and C-terminal tagging strategies, a TEV protease cleavage site can be added beside the flexible linker, which allows the removal of the Tag from the protein to provide flexibility for downstream experiments.^4^^,^^26^

***Alternatives*:** In some instances (*e.g.,* GC-rich sequences) DNA synthesis may not be possible. In this case, HAs can be obtained as double-stranded DNA fragments and cloned via Gibson assembly or amplified directly from genomic DNA (described later in Major Step “Cloning of the HDR plasmid via Gibson assembly”). The HAs will be cloned into the HDR plasmid using Gibson assembly; thus, they will need to contain a sequence of 20-25 bp on both sides that overlap with the HDR plasmid backbone and the Tag sequence (Figure 10A).

## Key resources table

**Table:** 

REAGENT or RESOURCE	SOURCE	IDENTIFIER
**Antibodies**		

Anti-ALFA mouse (1:1,000 dilution)	NanoTag	N1582
Anti-HA mouse (1:1,000 dilution)	Cell Signaling Technology	2367S
Anti-V5 rabbit (1:1,000 dilution)	Cell Signaling Technology	13202
Anti-Halo mouse (1:1,000 dilution)	Promega	G921
Anti-SNAP rabbit (1:1,000 dilution)	GenScript	A00684
HRP anti-rabbit antibody (1:5,000 dilution)	Abcam	AB6721
HRP anti-mouse antibody (1:5,000 dilution)	Abcam	AB6789

**Bacterial and virus strains**		

Top10 *E. coli* competent cells	Life Technologies	C404003

**Chemicals, peptides, and recombinant proteins**		

FastDigest BpiI enzyme	Thermo Scientific	FD1014
Dulbecco’s modified Eagle’s medium – high glucose (DMEM)	Thermo Fisher Scientific	41966029
Fetal bovine serum (FBS)	Corning	35-079-CV
FluoroBrite DMEM	Thermo scientific	A1896701
FuGENE(R) HD transfection reagent	Promega	E2311
Geneticin disulfate (G418)-solution	Gibco	11811-098
GlutaMAX	Gibco	35050061
HEPES PUFFERAN	Carl Roth	9105.2
Opti-MEM	Thermo Fisher Scientific	31985070
Penicillin-streptomycin	Fisher Scientific	DE17-602E
Phosphate-buffered solution (PBS)	Lonza	17-156F
Bovine serum albumin (BSA)	Carl Roth	8076.4
20X Bolt MES SDS running buffer	Life Technologies	B000202
ROTI Fair TG-Western	Carl Roth	1276.1
Puromycin	Life Technologies	A1113803
Quick calf intestinal alkaline phosphatase (CIP)	New England Biolabs	M0491S
Trypsin-EDTA solution	Lonza	17-161E
T4 DNA ligase	New England Biolabs	M0202S
Deoxynucleotide (dNTP)	New England Biolabs	N0447S
Phusion polymerase	Thermo Scientific	F530S
Bolt 4%–12% bis-tris gels	Thermo Scientific	NW04120BOX

**Critical commercial assays**		

Wizard Plus SV minipreps DNA purification system	Promega	A1460
QIAquick gel extraction kit	QIAGEN	28706
QIAquick PCR purification kit	QIAGEN	28106
QIAquick DNA extraction kit	Lucigen	QE09050
SuperSignal West Pico Plus	Fisher Scientific	34579

**Experimental models: Cell lines**		

HeLa CCL-2	ATCC	N/A

**Oligonucleotides**		

Designed guide RNA/primer sequences	Integrated DNA Technologies	N/A

**Recombinant DNA**		

pBS598 EF1alpha-EGFPcre	Le et al.^32^	Addgene plasmid #11923
pX330-U6-Chimeric_BB-CBh-hSpCas9	Cong et al.^8^	Addgene plasmid #42230
AP1muA single guide RNA	Stockhammer et al.^3^	Addgene plasmid #230030
AP1muA-TurboID-polyA-G418 HDR donor plasmid	Stockhammer et al.^3^	Addgene plasmid #227734
AP1muA-Halo-ALFA-polyA-G418 HDR donor plasmid	Stockhammer et al.^2^	Addgene plasmid #229681
AP1muA-mStayGold-polyA-Hygromycin HDR donor plasmid	Stockhammer et al.^2^	Addgene plasmid #229679
AP1muA-SNAP-V5-polyA-Puro HDR donor plasmid	Stockhammer et al.^2^	Addgene plasmid #229680
Rab11 single guide RNA	Stockhammer et al.^2^	Addgene plasmid #229683
LoxP-G418-LoxP-3xALFA-Halo-Rab11 HDR donor plasmid	Stockhammer et al.^2^	Addgene plasmid #229676
LoxP-Puro-LoxP-3xV5-SNAP-Rab11 HDR donor plasmid	Stockhammer et al.^2^	Addgene plasmid #229677
LoxP-Hygromycin-LoxP-HA-mStayGold-Rab11 HDR donor plasmid	Stockhammer et al.^2^	Addgene plasmid #229678
LoxP-G418-LoxP-TurboID-Rab11 HDR donor plasmid	This paper	Addgene plasmid #230026

**Software and algorithms**		

Benchling	Benchling	www.benchling.com
SnapGene Viewer	SnapGene	www.snapgene.com

**Other**		

Glass bottom dish 35 mm with 14 mm micro-well, #1.5 cover glass	IBL	D35-14-1.5-N
Janelia Fluor 646 HaloTag ligand	Promega	GA1120
SNAP-Cell 647-SiR ligand	New England Biolabs	S9102S
Janelia Fluor JFX650 HaloTag ligand	Promega	HT1070

## Materials and equipment

### Buffers for cloning

**Table:** Annealing buffer

Reagent	Final concentration
Tris, pH 7.5–8.0	10 mM
NaCl	50 mM
EDTA	1 mM

This solution should be made in autoclaved water and can be stored at 20°C for up to 1 year.

**Table:** TE buffer

Reagent	Final concentration
Tris-HCl pH 6.8	10 mM
EDTA pH 8	1 mM

This solution should be made in autoclaved water and can be stored at 20°C for up to 1 year.

### Media for cell culture

**Table:** Growth medium

Reagent	Final concentration	Amount
FBS	10%	50 mL
Penicillin/streptomycin	1%	5 mL
DMEM	N/A	445 mL

This solution needs to be made in sterile conditions and stored at 4°C.

**Table:** Live-cell imaging solution

Reagent	Final concentration	Amount
GlutaMAX (100X)	1%	5 mL
HEPES	2%	10 mL
FBS	10%	50 mL
FluoroBrite DMEM	N/A	435 mL

Media needs to be made in sterile conditions and stored at 4°C.

### Buffers for western blot

**Table:** 2X Laemmli Buffer

Reagent	Final concentration
SDS	4%
Glycerol	20%
Bromophenol blue	0.02%
Tris-HCl pH=6.8	120 mM
BME	5%

This solution can be made as a stock without BME and stored at 20°C (we recommend adding BME on the day of the sample preparation, once BME is added the solution can be stored at −20°C).

**CRITICAL:** BME should only be opened under a fume hood.

**Table:** PBST

Reagent	Final concentration
Tween 20	0.5%
1X PBS	N/A

This solution can be stored at 20°C.

**Table:** Blocking buffer

Reagent	Final concentration
Milk powder	5%
BSA	1%
PBST	N/A

This solution must be made freshly.

***Alternatives:*** For simplicity, this protocol is written for editing of HeLa cells, and plasmid transfection was carried out with FuGENE. Other cell lines can be successfully edited if efficient transfection can be achieved via any transfection methods. We have successfully edited various cell lines including HAP1, Jurkat, RPE-1, MCF7, MCF10, T84, Caco2, U-2 OS, RBL-2H3, LUVA^1^^,^^2^^,^^5^^,^^17^ using a NEPAgene NEPA21 electroporation machine.

***Alternatives:*** Substitute any molecular biology reagents successfully used in your laboratory (*e.g.,* polymerase, DNA extraction kits, chemically competent cells, etc.).

***Alternatives:*** Other antibiotics have successfully been used by our laboratories (Zeocin, Blasticidin). Many cell lines may have been immortalized with plasmids harboring antibiotic resistance cassettes (*e.g.,* RPE-1 are resistant to Hygromycin and Puromycin). Always check which antibiotics work for your cell line of choice.

***Alternatives:*** Double-KIs can be created simultaneously (transfecting wild type (WT) cells with two gRNAs and two HDR donor plasmids, each targeting a different locus) or sequentially (transfect an existing single CRISPR KI cell line with a gRNA and HDR donor plasmid, which targets a different locus). To ensure an efficient selection of edited cells, we recommend using a different antibiotic resistance cassette for each HDR donor plasmid (*e.g.*, Puromycin, G418, Hygromycin). Cells can then be treated with optimized concentrations of both antibiotics, which may improve efficiency. We have successfully gene-edited up to three loci using different antibiotic resistance cassettes. In our experience the final efficiency will be the product of individual KI efficiencies.

## Step-by-step method details

### Cloning of the gRNA in the pX330 plasmid

**Timing: 3–5 days**

In this section we will describe the cloning of a gRNA into the pX330-U6-Chimeric_BB-CBh-hSpCas9 plasmid from Addgene (#42230) (Figure S2).

***Note:*** This plasmid allows for the simultaneous expression of the Cas9 and the chosen guide. The single-stranded DNA oligonucleotides (oligos) that were designed and ordered as described in the previous section (Preparation Steps 1–12) will be annealed and cloned into the pX330 plasmid linearized with the restriction enzyme BpiI.

1. Prepare LB-agar plates containing ampicillin (100 μg/mL).
2. Order pX330-U6-Chimeric_BB-CBh-hSpCas9 plasmid from Addgene (#42230).

***Note:*** This plasmid allows for dual expression of the *Streptococcus pyogenes* Cas9 and the single gRNA (sgRNA) upon transfection of human cell lines.

3. Anneal guide oligos.
  a. Resuspend the oligos in nuclease-free water to a concentration of 1 mg/ml.
  b. For annealing, mix:
    i. 4 μL sense oligo.
    ii. 4 μL anti-sense oligo.
    iii. 42 μL annealing buffer.
  c. Using a PCR thermocycler, heat the oligo mixture to 95°C for 2 min. and decrease the temperature by 1 °C per min. to 25 °C.
    **Pause point:** Annealed oligos can be stored at −20 °C for up to a year, depending on the supplier recommendations (optional).
4. Purification and enzymatic digestion of pX330.
  a. Transform Top10 *Escherichia coli* (*E. coli*) with the pX330 plasmid and plate on a LB-Ampicillin agar plate.
  b. The following day pick a colony from the plate and inoculate 5 mL of LB medium containing Ampicillin (100 μg/mL), incubate 18 h. at 37 °C (shaking).
  c. The following day, proceed with plasmid extraction using the Wizard Plus SV Minipreps DNA Purification System according to the manufacturer’s protocol (https://www.promega.de/-/media/files/resources/protcards/wizard-plus-sv-minipreps-dna-purification-system-quick-protocol.pdf?rev=8293c52acfca4de495f1df90a7753d50&sc_lang=en).
    ***Note:*** Purification of the pX330 plasmid can also be done in Maxiprep format for multiple uses.
  d. Digest 2 μg of the pX330 plasmid with 2 μL FastDigest BpiI in the provided digestion buffer (1X final concentration) in a total volume of 50 μL TE buffer for 60 min. at 37 °C.
    ***Note:*** As a control, collect 1 μL of the sample before addition of the enzyme. Load 1 μL of digestion mix (before enzyme addition and after 60 min. on a 1% agarose gel. If supercoiled/undigested plasmid is visible on the gel in the 60 min. digestion condition, extend incubation time or troubleshoot your reagents.
  e. Load the entire digestion mixture on a 1% agarose gel for gel purification.
    ***Note:*** Use a comb with larger wells when casting the gel, allowing electrophoresis of the whole digestion reaction (48 μL).
  f. Excise the DNA fragment corresponding to ∼8.5 kb from the gel using a scalpel and extract the DNA from the gel using a QIAquick Gel Extraction Kit. Elute the purified plasmid in 40 μL of provided elution buffer.
    **CRITICAL:** Do not dephosphorylate the digested plasmid when using non-phosphorylated oligos as donor phosphate groups are needed for the ligation reaction.
    ***Alternatives:*** Phosphorylated annealed oligos can be cloned into a dephosphorylated, linearized pX330 plasmid.
    **Pause point:** The digested and purified plasmid can be stored at −20 °C for future use (optional).
5. Ligation of annealed oligos into the linearized pX330 plasmid.
  a. Set up the ligation reaction as shown in Table 1 at 20 °C for 2 h. or 4 °C for 18 h.

**Table 1:** Ligation of linearized pX330 with the annealed gRNA oligos

	Linearized pX330 (from Step 4)	Annealed oligos (from Step 3)	T4 DNA ligase (400,000 units/ml)	10X Ligase buffer	TE buffer
Control 1	1 μL (∼50 ng)	–	–	2 μL	Fill up to 20 μL
Control 2		–	1 μL		
Ligation		2 μL (∼320 ng)	1 μL		

Note: Control 1 tests the amount of undigested plasmid, control 2 will reveal the amount of self-ligating plasmid.

6. Transformation of the ligation mixture into competent TOP10 *E. coli*.
  a. Remove a 50 μL aliquot of Top10 *E. coli* from the −80^o^C freezer and thaw on ice for ∼10 min.
  b. Add 5 μL of the ligation mixtures (from Step 5) to an aliquot of Top10 *E. coli.* Gently flick the tube a couple of times to mix the plasmid and the competent bacteria. Then place the tube back on ice for ∼15 min.
  c. Transform *E. coli* by heat-shocking the DNA-bacteria mixture at 37 °C for 3 min.
  d. Add 500 μL LB medium (without antibiotic) to each tube, incubate for 15 min. at 37 °C (shaking).
  e. Plate bacteria on LB agar plates containing 100 μg/mL Ampicillin, incubate LB-agar plates at 37 °C for 18 h.

7. Screening of clones by Sanger sequencing to confirm ligation of the gRNA.
  a. Pick 2 colonies and inoculate 2 x 5 mL LB medium containing ampicillin (100 μg/mL) and incubate for 18 h. at 37 °C (shaking).
    ***Note:*** A successful ligation is indicated by a few or no colonies growing on the control plates and many colonies on the ligation plate.
  b. The following day, extract plasmid DNA using a Wizard Plus SV Minipreps DNA Purification System and measure the DNA concentration.
  c. Send 2 clones for Sanger sequencing using a U6 se primer (5′CAAGGCTGTTAGAGAGATAATTGGA 3′).
    ***Note:*** This primer binds the U6 promoter region upstream of the gRNA cloning site allowing to confirm insertion of the gRNA sequence into pX330 (Figure S2).

### Generation of HDR donor plasmids for N-terminal tagging

**Timing: 3–5 days**

Here, we will describe the single cloning step required to generate HDR donor plasmids for an N-terminally-tagged target.

***Note:*** As described in Preparation Steps 13–14, you should have synthesized an HDR donor plasmid containing the left homology arm (LHA), followed by the NheI and BamHI restriction sites, a glycine-serin linker (L), and the right homology arm (RHA) [LHA-NheI-NNNNN-BamHI-L-RHA; NNNNN are random bases to enable efficient restriction digest]. The desired Tag and antibiotic resistance cassette insert combination can be simply copied and pasted from the N-terminal FAB-CRISPR plasmid collection (Addgene ID: 229676-8 and 230026; Figure 11) as a NheI-BamHI insert.

8. Prepare LB-agar plates containing ampicillin (100 μg/mL).
9. Order plasmids from Addgene and make DNA Minipreps.
10. Linearization of the synthesized HDR donor plasmid.
  a. Digest 2 μg of your HDR donor plasmid with 2 μL each of NheI-HF and BamHI-HF in CutSmart buffer (1X final concentration) in a total volume of 50 μL TE buffer for 60 min at 37 °C.
    ***Note:*** To assess digestion efficiency, collect 1 μL of the sample before and after digestion. Load the samples on a 1% agarose gel. If supercoiled/undigested plasmid is visible on the gel in the digested sample, extend incubation time or troubleshoot your reagents.
  b. Add 1 μL Quick CIP (5000 units/mL) to the reaction and incubate at 37 °C for 30 min.
    ***Note:*** Quick CIP removes phosphate groups from the ends of the linearized plasmid, preventing re-ligation.
  c. Clean up the digestion mix to remove the enzymes using the QIAquick PCR Purification Kit, elute in 40 μL of the provided elution buffer.
    **Pause point:** The digested and purified plasmid can be stored at −20 °C for future use (optional).
11. Generation of the N-terminal FAB-CRISPR DNA fragment.
  a. Amplify the FAB-CRISPR plasmid of choice as described in Step 6a-e (see Figure 11 for an overview).
  b. Digest 4 μg of your FAB-CRISPR plasmid of choice with 2 μL each of NheI-HF and BamHI-HF in CutSmart buffer (1X final concentration) in a total volume of 50 μL TE buffer for 60 min. at 37 °C.
    ***Note:*** As a control, collect 1 μL of the sample before and after digestion. Load the samples on a 1% agarose gel. One should expect to see a higher molecular weight band corresponding to the plasmid backbone (3671 bp) and a lower molecular band corresponding to the FAB-CRISPR insert of choice (for information about the sizes of the various inserts see Figure 11). Troubleshooting 1.
  c. Load the whole reaction on a 1% agarose gel for subsequent gel purification.
    ***Note:*** Use a comb with larger wells when casting an agarose gel, allowing electrophoresis of the whole digestion reaction (48 μL).
  d. Excise the desired DNA fragment (see Figure 11 for expected fragment sizes) from the gel using a scalpel and extract the DNA from the gel using the QIAquick Gel Extraction Kit and elute in 40 μL of the provided elution buffer.
    ***Note:*** When using the FAB-CRISPR insert LoxP-G418-LoxP-V5-TurboID excise both DNA fragments (corresponding to the G418 AB^R^ and the Tag; see Figure 11B).
    **Pause point:** The digested and purified fragments can be stored at −20 °C for future use (optional).
12. Ligation of the N-terminal FAB-CRISPR insert into the HDR donor plasmid.
  a. Set up a ligation reaction as shown in Table 2. Ligations can be carried out at 20 °C for 2 h. or 4°C for 18 h.

**Table 2:** Ligation of FAB-CRISPR fragments into a linearized HDR donor plasmid

	Linearized HDR (from step 10)	FAB-CRISPR Fragment (from step 11)	T4 DNA ligase (400,000 units/ml)	10X ligase buffer	TE buffer
Control 1	1 μL (∼50 ng)	–	–	2 μL	Fill up to 20 μL
Control 2		–	1 μL		
Ligation		2 μL (∼200 ng)	1 μL		

Note: For the ligation of the two fragments resulting from digestion of the LoxP-G418-LoxP-V5-TurboID insert (see Figure 11B), we recommend using 25 ng of linearized HDR plasmid and 1 μg DNA for each fragment.

13. Continue with transformation and plasmid amplification, as described in Steps 6a-e.

***Note:*** Plate your transformed cells onto an antibiotic-containing plate that corresponds to the resistance cassette in your chosen backbone.

**Figure 12:**
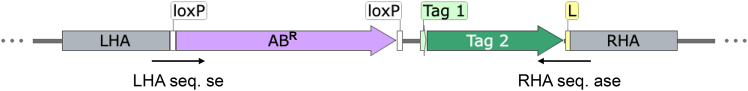
Schematic of a target HDR donor plasmid for N-terminal tagging and suggested sequencing (seq) primers Left homology arm (LHA), right homology arm (RHA), AB^R^ (antibiotic resistance cassette), se (sense), ase (anti-sense), L (glycine-serine linker). Map is visualized using the SnapGene software.

14. Confirming successful cloning via restriction digest and Sanger sequencing.
  a. Pick 3 colonies and inoculate 2 x 5 mL LB medium containing ampicillin (100 μg/mL) and incubate for 18 h. at 37 °C (shaking).
    ***Note:*** Successful ligation is indicated by a few or no colonies growing on the control plates and many colonies on the ligation plate.
  b. The following day extract plasmid DNA using a Wizard Plus SV Minipreps DNA Purification System and measure the DNA concentration.
  c. Digest 500 ng of each DNA Miniprep (from Step 14b) with 0.1 μL each of BamHI-HF and NheI-HF in a total volume of 10 μL of TE buffer.
  d. Load digests of DNA Minipreps from different clones on a 1% agarose gel.
  e. Select the clones which display the correct digestion pattern (see Step 14d) for Sanger sequencing with a sense and an anti-sense primer that anneal with the LHA and RHA sequence, respectively.
    ***Note:*** Design the sequencing primer to align ∼80 bp upstream (sense) and downstream (anti-sense) from the target sequence for optimal sequencing results (Figure 12).
    ***Alternatives*:** Full-length sequencing for plasmid verification is now performed by many sequencing providers.

**Figure 11:**
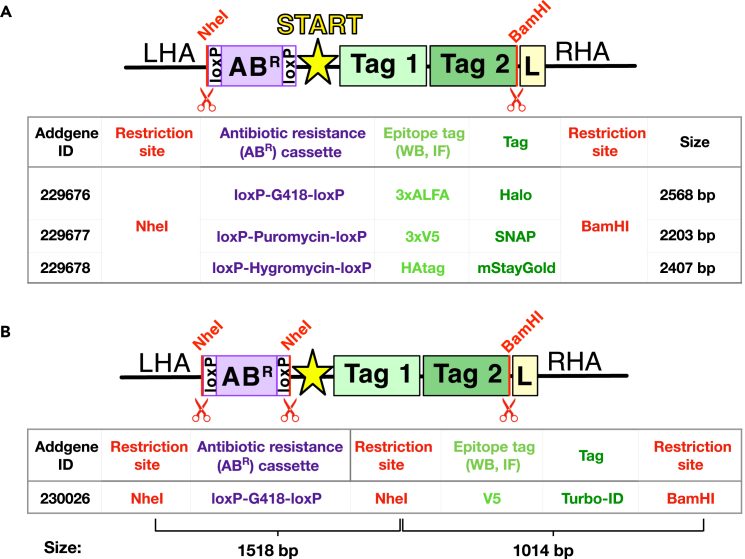
Overview of the available N-terminal FAB-CRISPR inserts (A and B) For N-terminal fusions, the resistance cassette sequence is flanked by LoxP sites, allowing excision via EGFP-Cre recombinase. The FAB-CRISPR insert LoxP-G418-LoxP-V5-TurboID (B) has an additional NheI site necessitating purification and ligation of two fragments. Left homology arm (LHA), right homology arm (RHA), AB^R^ (antibiotic resistance cassette), monomeric StayGold (mStayGold), L (glycine-serine linker). Not drawn to scale.

### Generation of HDR donor plasmids for C-terminal tagging

**Timing: 3–5 days**

Here, we will describe the single cloning step required to generate HDR donor plasmids for a C-terminally-tagged target.

**Figure 13:**
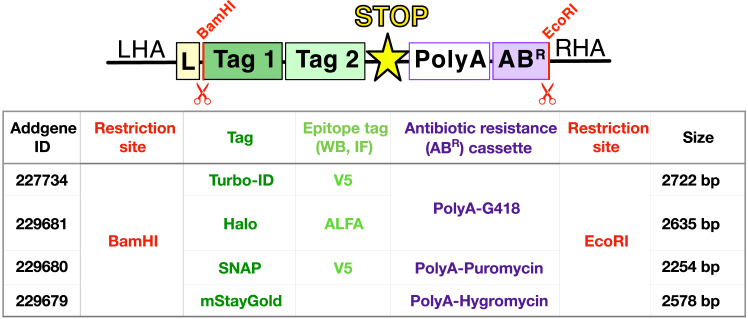
Overview of the available C-terminal FAB-CRISPR inserts The presence of an exogenous SV40 polyA sequence downstream of the Tag eliminates the need to excise the resistance cassette while allowing expression of the endogenous target. Left homology arm (LHA), right homology arm (RHA), AB^R^ (antibiotic resistance cassette), monomeric StayGold (mStayGold), L (glycine-serine linker). Not drawn to scale.

***Note:*** As described in Preparation Steps 13–14, you should have synthesized an HDR donor plasmid containing: the left homology arm (LHA), followed by a glycine-serine linker (L), a BamHI restriction site, an EcoRI restriction site and the right homology arm (RHA) [LHA-L-BamHI-NNNNN-EcoRI-RHA; NNNNN are random bases to enable restriction digests]. The desired Tag and antibiotic resistance cassette combination can be copied and pasted from the C-terminal FAB-CRISPR plasmids collection (ID: 229679-81, 227734; Figure 13). The desired insert is simply excised as a BamHI-EcoRI fragment for insertion into your newly synthesized HDR donor plasmid.

15. Linearization of the synthesized HDR donor plasmid, generation of the FAB-CRISPR insert and ligation.

This can be done as previously described in Steps 10–13 by simply using BamHI-HF and EcoRI-HF as restriction enzymes.

***Note:*** One should expect to see a higher molecular weight band corresponding to the AP1μA HDR plasmid backbone (4379 bp) and a lower molecular band corresponding to the FAB-CRISPR insert of choice (for information about the size of the various inserts see Figure 13).

**Figure 14:**
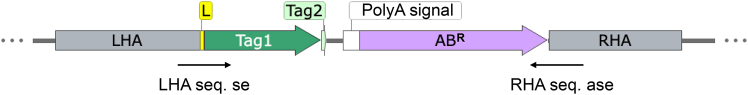
Schematic of a target HDR donor plasmid for C-terminal tagging and suggested sequencing (seq) primers Left homology arm (LHA), right homology arm (RHA), AB^R^ (antibiotic resistance cassette), se (sense), ase (anti-sense), L (glycine-serine linker). Map is visualized using the SnapGene software.

16. Confirm successful cloning via restriction digest and Sanger sequencing.
  a. Prepare and digest your Minipreps as described in Steps 14a-e but using BamHI-HF and EcoRI-HF.
  b. Select the clones which display the correct digestion pattern (see Figure 13).
  c. Select the Minipreps showing the correct digestion pattern for Sanger sequencing with a sense and an anti-sense primer that align with the LHA and RHA sequence, respectively (see Figure 14).

***Alternatives*:** Full-length sequencing for plasmid verification is now performed by many sequencing providers.

### Customizing FAB-CRISPR HDR donor plasmid

17. To modify the provided FAB-CRISPR inserts, the AB^R^ and Tags can be interchanged/switched via XhoI (N-terminal HDR donor plasmids) or NotI (C-terminal HDR donor plasmids) through copy and paste as previously described in Steps 8–16.

***Note:*** An overview of the possible fragments obtained upon digest is shown in Figures S3A and S3B.

18. Custom inserts can be generated through PCR by using a DNA template encoding the desired sequence. For this design two primers with unique restriction sites that each contain at least 20 nucleotides overlap with the beginning (sense primer) or end (anti-sense primer) of the desired insert encoded in the DNA template (Figure S3C).

**CRITICAL:** When exchanging Tags make sure to include a start codon at the beginning (N-terminal) or a stop codon at the end (C-terminal) of the Tag.

### Cloning of the HDR plasmid via Gibson assembly

**Timing: 5 days**

Here we will describe step-by-step how to construct an HDR donor plasmid from double-stranded DNA fragments (dsDNA) fragments via Gibson assembly. First, left and right homology arms are either synthesized as dsDNA as previously described in “Designing a homology-directed repair donor (HDR) plasmid for N- or C-terminal tagging” or amplified via PCR from genomic DNA. Second, a Tag fragment is generated via PCR. Finally, the three fragments (LHA-Tag-RHA) are assembled into a vector via Gibson assembly. The generated HDR donor plasmid can then be then used for transfection and editing, as laid out from Major Step “Transfection of editing reagents into HeLa cells”. The following steps are described in detail below.

19. Design of the left and right homology arms.
  a. For each target gene, two dsDNA fragment encoding the right and left homology arms (HA) flanking the tag insertion site must be designed using Benchling (see “Designing a homology-directed repair donor (HDR) plasmid for N- or C-terminal tagging” described previously).
    ***Note:*** We have successfully used HA, which are of lengths 250–450 bp downstream/upstream of the START codon (N-terminal fusion) or downstream/upstream from the STOP codon (C-terminal fusion).
    **CRITICAL:** The HA will be cloned within the HDR vector using Gibson assembly and must contain a sequence of 20–25 bp on both sides that overlap with the HDR plasmid backbone and the Tag sequence.
  b. Choose a vector for cloning the HDR donor.
    ***Note:*** Ideally, the vector should be a high copy number plasmid containing a multi cloning site (MCS) for restriction digestion. We recommend the pFastBac Dual vector (Thermo Fisher Scientific #10712024) but other high-copy plasmids are suitable for HDR cloning, including pUC cloning vectors.
  c. Synthesis the HA as a double-stranded DNA fragment from a nucleic acid supplier.
    ***Note:*** In some instances (*e.g.,* GC-rich sequences) DNA synthesis is not achievable. In this case, HA can be obtained through the amplification of genomic DNA using PCR.
    ***Note:*** When using pFastBac Dual, we linearize the plasmid using the HpaI restriction enzyme (NEB) so the LHA will have 20–25 bp overlapping with the vector plasmid on the 5′ end of the HpaI cut site, and 20–25 bp overlapping with the Tag. Similarly, the RHA will have 20–25 bp on the 5′ end overlapping with the Tag, and 20–25 bp on the 3′-end of the HpaI cut site overlapping with the vector.
    ***Note:*** If the HA is PCR-amplified from genomic DNA, use SnapGene or analogous cloning software to design the amplification primers. The primers must contain 20–25 bp overlapping with the adjacent gene fragment or vector to be cloned using Gibson assembly.
  d. Perform an *in silico* Gibson assembly using cloning design software, such as SnapGene or Benchling.
    **CRITICAL:** For the N-terminal Tag, verify that the *in silico* assembled HDR plasmid contains a START codon immediately preceding the Tag. For the C-terminal, verify the presence of a STOP codon at the end of the Tag fragment.
    ***Alternatives:*** To overcome PCR-amplification of HA from genomic DNA when HAs are difficult to synthesize, we have had success shortening the HA to 300–400 bp. However, this strategy could reduce the efficiency of homology-directed repair.
    **CRITICAL:** If the gRNA does not span the Cas9 cleavage site, the Cas9 will cleave the HDR donor plasmid. To overcome this issue, insert a silent mutation(s) in the homologous donor sequence that will prevent the gRNA from binding.
20. Generation of the Tag fragment: Tag amplification and purification.
  a. Obtain the Tag sequence according to your KI strategy.
    ***Note:*** The plasmid encoding the N-terminal Halo Tag can be ordered from Addgene (#86843).
  b. Using a cloning software, design primers to amplify the Tag.
    ***Note:*** The sense primer will contain 20–25 bp on the 5′-end overlapping with the LHA on the 3′- end. For the anti-sense primer, a 3′-end overlap is not needed if included in the 5′-end of the RHA.
    ***Note:*** Primers with 20–25 bp overlap with the HA are not required if the overlapping sequences with the Tag are included when designing the HA.
    ***Note:*** For optimal amplification, the primers should have similar T_m_ for efficient PCR.
  c. Perform the PCR and purify the product after electrophoresis on 1% agarose gel using a gel extraction kit and elute in at least 30 μL elution buffer.
  d. Measure the concentration of the purified Tag PCR product.
  e. Dilute the purified Tag PCR product to a concentration of ∼20 ng/μL.

21. Linearization of the vector: pFastBac Dual Digestion and Purification.
  a. Digest the pFastBac Dual plasmid using HpaI restriction enzyme by assembling the reaction listed in Table 3 in a 200 μL PCR tube, incubate for 60 min at 37 °C in a thermocycler.
    ***Note:*** In the case of a different vector, adjust the protocol accordingly to the restriction enzyme used.
    **CRITICAL:** Because HpaI generates blunt DNA ends, dephosphorylation of 5′-ends of vector DNA is critical to prevent re-ligation of the backbone.

**Table 3:** Digestion reaction mix

Component	Volume (μL)
pFastBac (1 mg/mL)	10
10x CutSmart Buffer	5
HpaI-HF	3
ddH_2_O	32
Total	50
  b. Add 2 μL of Quick CIP to the enzyme digestion preparation and incubate at 37 °C for 30 min.
    **CRITICAL:** Perform the dephosphorylation step immediately after HpaI digestion using Quick CIP.
  c. Stop the reaction at 80 °C for 2 min.
  d. Purify the digested vector after electrophoresis on 1% agarose gel using a gel extraction kit and elute in at least 25 μL elution buffer.
    ***Note:*** In the electrophoresis, run a small amount of undigested pFastBac vector alongside the digested vector as a control to confirm digestion.
  e. Measure the concentration of the digested vector and dilute it to 50 ng/μL.

22. Assembly of the HDR template from dsDNA fragments.
  a. Dilute the HA in TE Buffer to a concentration of 10 ng/μL following the protocol provided by the supplier.
  b. Set up the Gibson assembly reaction, using the Gibson Assembly MasterMix or the NEBuilder HiFi DNA Assembly Master Mix from NEB (#E2611 or #E2621).
    ***Note:*** For an efficient Gibson assembly reaction, you want to use an excess molar ratio of each insert relative to the starting concentration of the DNA backbone. We have had much success using a 1:3 molar ratio between vector and inserts. For calculating the volume of each insert needed for the Gibson Assembly reaction, use the NEBioCalculator (https://nebiocalculator.neb.com/#!/ligation).
    ***Note:*** As a negative control, omit all the DNA inserts and compensate the volume with ddH_2_O.
  c. Prepare the reaction listed in Table 4 in a 200 μL PCR tube, gently mix and incubate at 50 °C for 1 h. in a thermocycler, and then ramp down to 4°C.

**Table 4:** Gibson assembly pipetting scheme

Component	Volume (μL)
Digested pFastBac (50 ng/μL)	1
LHA (10 ng/μL)	1
RHA (10 ng/μL)	1
Tag (20 ng/μL)	2.5
Gibson Mastermix (2X)	10
ddH_2_O	4.5
Total	20
  d. Following incubation, store samples on ice or at −20 °C for subsequent transformation.

23. Transformation of the assembled HDR template.
  a. Add 5 μL of the Gibson assembly mixture to 50 μL *E. coli* TOP10, incubate for 25 min. on ice.
  b. Heat-shock the cells at 42 °C for 45 s.
  c. Incubate on ice for 3 min.
  d. Plate the cells on ampicillin-containing agar plates, incubate at 37 °C for 18 h.

**CRITICAL:** Count the colony numbers. Usually, an efficient Gibson assembly will result in the absence of colonies in the control plate and 10-50 colonies in the assembly plate. If the control and assembly plate have a similar number of colonies, repeat the Gibson assembly. Colonies in the control plate may also indicate incomplete restriction digestion of the vector plasmid. In that case, it is recommended to repeat the restriction digestion and subsequent purification.

24. Restriction digestion for verifying correct assembly of the HDR donor plasmid.
  a. Select 2–5 colonies and inoculate 5 mL LB Broth containing ampicillin (100 mg/mL) and place in a bacterial shaker for 18 h. (shaking).
  b. The following day, extract plasmid from *E. coli* using a Miniprep plasmid extraction kit and measure the DNA concentration.
  c. Digest the pFastBac Dual plasmid using a restriction enzyme by assembling the reaction listed in Table 5.
    ***Note:*** When using the pFastBac, we have used BamHI enzyme, which cuts both the vector backbone and at a BamHI site in both the N- and C-Terminal Halo Tag sequence between the HA. In the case of a different vector or Tag, adjust the protocol accordingly to the restriction enzyme used.

**Table 5:** Digestion reaction mix

Component	Volume (μL)
pFastBac (1 mg/mL)	1
10x CutSmart Buffer	2
BamHI	1
ddH_2_O	16
Total	20
  d. Run the PCR product on a 1% agarose gel and verify that the digested product contains two discrete bands.
    ***Note:*** The lack of HDR insertion (likely annealed backbone) will show a single band.
  e. Design and order primers for sequencing the entire HDR DNA that you have cloned into pFastBac Dual.
  f. Send the HDR plasmid for sequencing and confirm by importing Sanger sequencing results into SnapGene and aligning to your *in silico* Gibson assembled plasmid.
    ***Alternatives:*** Instead of restriction enzyme digestion, the correct assembly of the HDR donor plasmid can be verified by colony PCR (follow your polymerase of choice provider instructions). The amplification of a fragment within the LHA-Tag-RHA will indicate the presence of the HDR sequence. As an alternative to multiple Sanger sequencing, full-length plasmid sequencing is now widely available.

### Transfection of editing reagents into HeLa cells

**Timing: 3 days**

For editing a gene of interest, the generated HDR donor plasmid (containing the Tag of interest and the antibiotic resistance cassette) and the pX330 plasmid (encoding for the Cas9 and the gRNA) are co-transfected into HeLa cells.

**CRITICAL:** Maintain HeLa cells in growth medium at 37 °C with 5% CO_2_.

**Figure 15:**
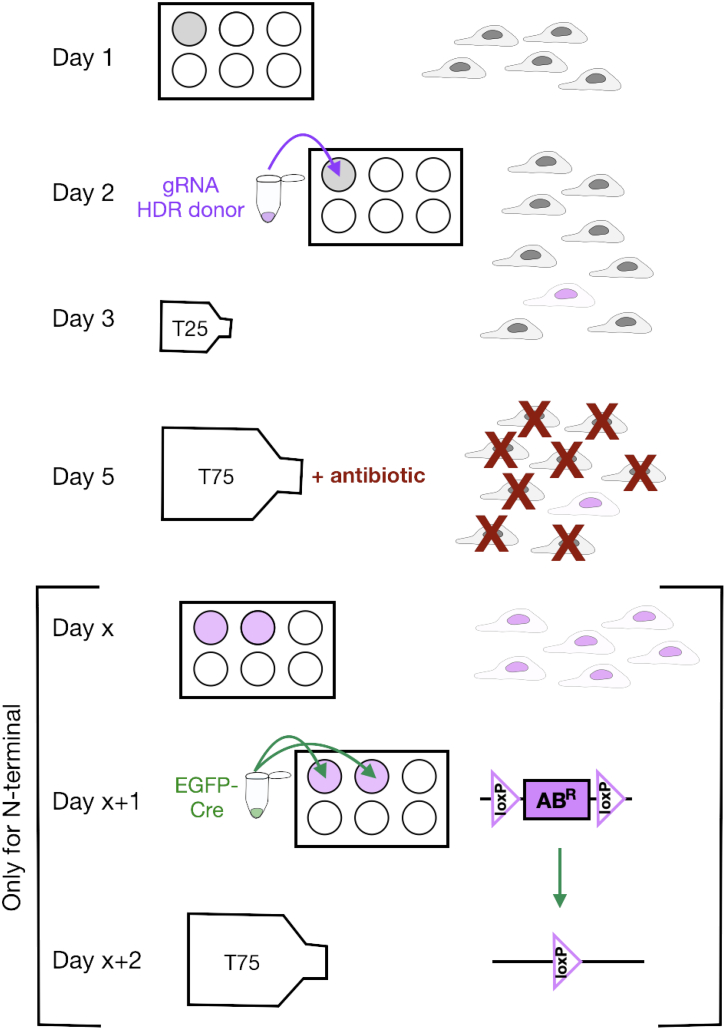
Timeline for the generation of a FAB-CRISPR gene-edited cell line Positively edited KI cells (shown in purple) integrate an antibiotic resistance cassette into their genome, in addition to the desired Tag. KI cells can be enriched over WT cells (shown in gray) through antibiotic selection (red). When knocking in a Tag at the N-terminus of a protein of interest, the resistance cassette (flanked by LoxP sites) needs to be excised by transfecting an EGFP-Cre recombinase-encoding plasmid (green) after selection and regrowth of the cells (Day x). Not drawn to scale.

***Note:*** A timeline of cell culture manipulations, from transfection (Step 25) to selection of positively edited cells (Major Steps “Selection of edited cells (C/N-terminal tagging)”) is shown in Figure 15.

25. Co-transfection of the FAB-CRISPR HDR donor and pX330 plasmids into HeLa cells with the transfection reagent FuGENE.
  a. Day 1: Seed cells for transfection.
    i. Seed 200 000 WT HeLa cells per well into a 6-well plate.
      ***Note:*** Cells should be ∼60%–70% confluent the following day.
  b. Day 2: Transfect cells with FAB-CRISPR constructs.
    i. Prepare a transfection mixture containing: 1 μg HDR donor plasmid and 1 μg pX330 plasmid in a total volume of 100 μL OptiMEM.
    ii. Vortex the mixture briefly, then add 6 μL of FuGENE (1:3 DNA:FuGENE ratio).
    iii. Vortex the transfection mixture containing FuGENE, spin down briefly, incubate for 5 min. at 20 °C.
    iv. Wash the cells 3X with 2 mL of pre-warmed PBS.
    v. Add 500 μL of pre-warmed OptiMEM to each well, then apply the transfection mixture dropwise.
      **CRITICAL:** Gently swirl the 6-well plate to ensure the transfection mixture is evenly distributed.
    vi. After ∼5 h. exchange the medium to 2 mL of pre-warmed growth medium, incubate for 18 h. at 37 °C with 5% CO_2_.
  c. Day 3: Transfer cells to a T25 flask.
    i. Wash each well with 2 mL of pre-warmed PBS.
    ii. Detach cells by adding 500 μL trypsin, incubate for 5 min. at 37 °C with 5% CO_2_.
    iii. Add 1 mL of of pre-warmed growth medium to the well, gently pipette up and down to resuspend the cells, and transfer them into a T25 flask in a total volume of 5 mL growth medium.
      ***Note:*** For troubleshooting or optimization of the transfection reaction, follow the manufacturer’s instruction (https://www.promega.de/-/media/files/resources/protocols/technical-manuals/101/fugene-hd-transfection-reagent.pdf).
      ***Note:*** When creating a double-KI, simultaneously transfect pX330 and HDR donor plasmids (*e.g.,* 2 HDR plasmids containing resistance cassettes conferring resistance to both Puromycin and G418) to edit both loci (0.5 μg of each plasmid) with 6 μL FuGENE. See the alternative section (page 14) for more details.

### Selection of edited cells (C-terminal tagging)

**Timing: 1–2 weeks**

Homology-directed repair (HDR) is a rare event. To enrich for cells that have undergone HDR and have been successfully edited, we take advantage of the antibiotic resistance cassette present on the HDR donor plasmids as this is integrated into the genome with the Tag. When editing a protein of interest at the C-terminus, we employ a FAB-CRISPR insert containing the desired Tag, followed by an exogenous polyA sequence and a resistance cassette (^16^; see Figure 13). Successfully edited cells will express the gene conferring antibiotic resistance and can be enriched by antibiotic selection which will be explained in this section.

**CRITICAL:** Before starting the antibiotic selection, determine the minimal antibiotic concentration that is sufficient to kill unedited HeLa cells. We have found a concentration of 1 mg/mL of G418, 1 μg/mL of Puromycin and 0.4 mg/mL of Hygromycin to be optimal for selection.

**CRITICAL:** Only select cells for the time required to successfully kill all WT non-edited cells to avoid selecting for cells with sustained expression of the selectable marker or where the selectable marker has been stably integrated into the genome.

26. Enrichment of gene-edited cells via antibiotic selection.
  a. Begin antibiotic treatment three days after transfection.
    ***Note:*** This gives the cells enough time to express the gRNA and Cas9 nuclease and edit the genome.
    i. Ensure cells are less than 50% confluent when first adding the antibiotic for optimal selection conditions.
    ii. Replace medium with growth medium containing the appropriate antibiotic (*e.g.*, G418, Puromycin, Hygromycin).
    iii. Maintain antibiotic pressure until only a few percent of the cells survive by exchanging the medium containing antibiotics every 2–3 days.
      ***Note:*** The antibiotic selection should be complete after one week (for G418) or 2–3 days (for Puromycin and Hygromycin). Troubleshooting 2 and 3.
  b. After antibiotic treatment, most cells should have died. The surviving cells will divide to form islands of positively-edited cells. Once these islands form, detach the cells and evenly redistribute cells in the same flask.
    i. Wash the T75 flask with 10 mL of pre-warmed PBS.
    ii. Detach the cells by adding 2 mL trypsin, incubate for 5 min. at 37 °C with 5% CO_2_.
    iii. Tap the flask gently to detach cells.
    iv. Add 10 mL of pre-warmed growth medium and gently pipette up and down to break up cell clumps.
      **Pause point:** After cells have recovered from antibiotic treatment, they can be frozen down with growth medium containing 10% DMSO for long-term storage (optional).

### Selection of edited cells (N-terminal tagging)

**Timing: 2–3 weeks**

For N-terminal tagging, an excisable resistance cassette, flanked by LoxP sites, is edited into the genome before the desired Tag.^31^ The resistance cassette must be excised for the edited protein to be expressed. After cells recover from antibiotic selection, they are transfected with a plasmid coding for EGFP-Cre (Addgene plasmid #11923^32^ to excise the resistance cassette.

For enrichment of gene-edited cells using antibiotic treatment refer to Step 26 of the previous Major Step, “Selection of edited cells (C-terminal tagging)”.

27. Excision of the resistance cassette with EGFP-Cre recombinase.
  a. Day 1: Seed antibiotic-selected cells for transfection.
    i. Seed 200 000 cells per well into a 6-well plate.
      ***Note:*** Cells should be ∼60%–70% confluent the following day.
      ***Note:*** We recommend seeding multiple wells which can be combined after transfection to obtain a confluent flask of cells more rapidly.
      ***Note:*** Keep some antibiotic-selected cells as a backup in case the EGFP-Cre transfection needs to be repeated.
  b. Day 2: Transfect cells with EGFP-Cre.
    i. Prepare a transfection mixture containing 2 μg EGFP-Cre in a total volume of 100 μL OptiMEM.
    ii. Vortex the mixture briefly and add 6 μL FuGENE (1:3 DNA:FuGENE ratio).
    iii. Vortex the transfection mixture containing FuGENE and spin it down briefly, incubate for 5 min. at 20 °C.
    iv. Wash the cells 3X with 2 mL of pre-warmed PBS.
    v. Add 500 μL of pre-warmed OptiMEM per well, then apply the transfection mixture dropwise.
      **CRITICAL:** Gently swirl the 6-well plate to ensure the transfection mixture is evenly distributed.
    vi. After ∼5 h. exchange to 2 mL of pre-warmed growth medium, incubate ON at 37 °C with 5% CO_2_.
  c. Day 3: Transfer cells into a T75 flask.
    i. Wash each well with 2 mL of pre-warmed PBS.
    ii. Detach the cells by adding 500 μL trypsin, incubate for 5 min. at 37 °C with 5% CO_2_.
    iii. Add 1 mL of pre-warmed growth medium in each well and resuspend the cells by gently pipetting up and down.
    iv. Combine cells into a T75 flask in a total volume of 10 ml growth medium.
      ***Note:*** To check the transfection efficiency of EGFP-Cre recombinase, examine the cells the next day using a cell culture LED-based fluorescence microscope equipped with a filter to detect green fluorescence; EGFP-Cre transfected cells will show green fluorescence in the nucleus.

### Verification of successfully edited cells

**Timing: 2 days (for step 28)**

**Timing: 2 days (for step 29)**

**Timing: 2 days (for step 30)**

**Timing: 2 days (for step 31)**

Gene-edited cells enriched via antibiotic selection can be validated through Western blotting, Sanger sequencing of the targeted genomic locus, and imaging.

***Note:*** When performing a Western blot analysis, it is important to use antibodies to detect both the Tag (*e.g.,* SNAP or Halo) but also an antibody specific for the protein of interest.

***Note:*** When clonal selection is not required, downstream experiments can be carried out with the mixed (homozygous and heterozygous) cell population.

**CRITICAL:** It is important to validate the localization and functionality of the endogenously tagged protein. For the Turbo-ID Kis, also validate whether the fusion protein can biotinylate proximal interactors (for more details see Cho et al.^33^).

28. Verification of endogenous tagging via Western blotting.
  a. Day 1: Seed cells for harvesting.
    i. Seed 200 000 gene-edited and 200 000 WT HeLa (negative control) cells in a 6-well plate.
      ***Note:*** Cells should be ∼80% confluent on the day of harvesting.
  b. Day 2: Sample preparation.
    i. Wash the wells twice with 2 mL of pre-warmed PBS.
    ii. Add 400 μL 2X Laemmli buffer containing 5% BME per well.
    iii. Scrape the wells with a cell scraper and transfer the cells into a 1.5 mL tube.
    iv. Boil the sample for 10 min. at 95 °C.
      **Pause point:** The boiled sample can be frozen down (−20 °C) for later use (optional).
  c. SDS Page and Western blotting.
    i. Vortex and load 25 μL of the sample per well in a pre-cast protein gel, alongside a molecular weight marker.
      ***Note:*** If the sample was frozen, boil and vortex before loading.
    ii. Run the SDS gel at 100–150 V until the marker has run sufficiently – this depends on the molecular weight of the protein of interest.
    iii. Transfer the proteins to a nitrocellulose membrane by wet blotting (1 h. at 350 mA).
      ***Note:*** To check the quality of the transfer, the membrane can be stained with Ponceau.
    iv. Block the membrane with blocking buffer for 1 h. at 20 °C (shaking).
    v. Wash the membrane for 15 min. with PBST (shaking).
    vi. Wash the membrane 3X for 5 min. with PBS (shaking).
    vii. Add the primary antibody for 1 h. at 20 °C or for 18 h. at 4 °C (shaking).
      ***Note:*** Dilute the desired antibody (*e.g*., anti-V5/ALFA/HA or anti-SNAP/Halo or an antibody specific for your protein of interest) antibodies 1:1000 in PBS supplemented with 0.02% sodium azide and 1% BSA.
    viii. Wash the membrane for 15 min. with PBST (shaking).
    ix. Wash the membrane 3X for 5 min. with PBST (shaking).
    x. Add an anti-mouse or anti-rabbit HRP-conjugated secondary antibody (depending on the primary antibody used) diluted 1:5000 in blocking buffer for 45 min. at 20 °C (shaking).
    xi. Wash the membrane for 15 min. with PBST (shaking).
    xii. Wash the membrane 3X for 5 min. with PBS (shaking).
    xiii. Develop the blot using the SuperSignal West Pico Plus chemiluminescent substrate. Troubleshooting 4, 5, and 8.
      ***Note:*** SuperSignal West Pico Plus is a high-sensitivity substrate which is ideal for detecting low abundance endogenously tagged proteins.
29. In-gel fluorescent detection of endogenously tagged proteins.
  a. Day 1: Seed cells for harvesting.
    i. Seed 60 000 cells in a 24-well plate in 500 μL of pre-warmed growth medium.
  b. Day 2: Labeling and in-gel fluorescent detection.
    i. Label Halo/SNAP tagged protein with 100 nM Janelia Fluor JFX650 HaloTag or SNAP-Cell 647-SiR ligands in 500 μL of pre-warmed growth medium, incubate for 30 min. at 37^o^C with 5% CO_2_.
    ii. Wash 3X with of pre-warmed PBS, and then exchange to 500 μL of pre-warmed growth medium, incubate at 37^o^C for 10 min. allowing any unbound dye to leach out of the cells into the medium.
    iii. Wash once with PBS and add 60 μL of 2X Laemmli buffer containing 5% BME.
    iv. Using a pipette, swirl the buffer around the well and transfer lysate to a 1.5 ml tube.
    v. Boil the sample at 95^o^C for 5 min. and load 25 μL onto a polyacrylamide gel alongside a molecular weight marker.
      ***Note:*** For optimal gel percentage refer to manufacturers website as it depends on the protein size (*e.g.,* Bolt 4%–12% Bis-Tris gels cover proteins in the range of 3,5 bis 260 kDa).
    vi. Run the gel at 200 V for ∼45 min. until the Laemmli buffer runs out of the bottom of the cassette.
    vii. Using the Cy5.5 filter on a Bio-Rad ChemiDoc, image fluorescence (1–60 s. depending on the expression of your tagged protein). Troubleshooting 5 and 8.

30. Sanger sequencing of the targeted genomic locus.
  a. Day 1: Seed cells for harvesting.
    i. Seed 10 000 gene-edited and 10 000 WT HeLa (negative control) cells in a 6-well plate.
  b. Day 2: Extract DNA.
    i. Wash the wells 3X with PBS.
    ii. Add 500 μL QuickExtract DNA Extraction Solution and transfer the cells using a cell scraper into a 1.5 mL tube.
    iii. Vortex the 1.5 mL tube for 15 s, incubate for 6 min. at 65 °C.
    iv. Vortex the 1.5 mL tube for 15 s, incubate for 2 min. at 98 °C.
      ***Note:*** For details see www.lucigen.com/docs/manuals/MA150E-QuickExtract-DNA-Solution.pdf.
  c. Genomic PCR.
    i. Set up a genomic PCR with the extracted genomic DNA and primers binding outside the HAs (see Table 6 for a detailed reaction; Table 7 for cycling conditions).

**Table 6:** PCR reaction mix

Reagent	Amount
5X Phusion Reaction buffer	5 μL
dNTPs	0.5 μL
Sense primer (10 mM)	1.25 μL
Anti-sense primer (10 mM)	1.25 μL
Template	25 ng
Phusion polymerase	0.25 μL
Millipore water	Up to 25 μL

**Table 7:** PCR cycling conditions

Steps	Temperature	Time	Cycles
Initial Denaturation	98 °C	5 min.	1
Denaturation	98 °C	30 s.	45
Annealing	x	1 min.	
Extension	72 °C	2 min.^a^	
Final extension	72 °C	5 min.	1
Hold	4 °C	∞	

x= Use recommended temperature of a Tm calculator (*e.g.*, https://tmcalculator.neb.com).

a Adjust to fragment size and according to polymerase manufacturer instructions.
  d. Gel purify the PCR fragment with a gel extraction kit and send the DNA for Sanger sequencing (see Figure 16). Troubleshooting 8.

**Figure 16:**
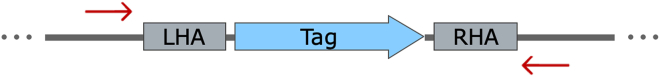
Schematic showing how to design PCR primers (red arrows) to amplify the targeted genomic locus Left homology arm (LHA), right homology arm (RHA). Not drawn to scale.

**Figure 17:**
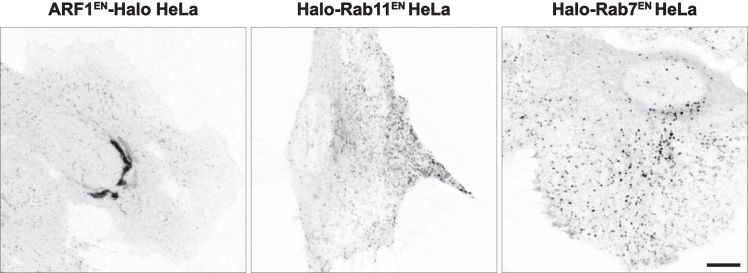
Example live-cell confocal images of different gene edited cell lines expressing endogenously tagged proteins localizing to different compartments ARF1^EN-^Halo (left, localizes to the Golgi and tubular endosomal compartments), Halo-Rab11 (middle, localizes to recycling endosomes) and Halo-Rab7 (right, localizes to late endosomes). HeLa cells labeled with JFX650 HaloTag before imaging on a confocal microscope. Scale bar is 10 μm. EN=endogenous.

31. Verification of the expression and localization of mStayGold-, Halo-, SNAP-tagged proteins with live-cell imaging.
  Gene-edited cells can be visualized via live-cell imaging using fluorescence (mStayGold) or by labeling the self-labeling enzymes SNAP and Halo with cell permeable substrates.
  ***Note:*** For the Turbo-ID KIs use a primary anti-V5 antibody in combination with a secondary antibody against the primary antibody carrying a fluorophore for immunofluorescence.
  a. Day 1: Seed cells for imaging
    i. Seed 160 000 gene-edited cells on a glass bottom 35 mm dish in a total volume of 2 mL growth medium.
      ***Note:*** For optimal imaging, ensure cells are ∼70% confluent the following day.
  b. Day 2: Stain Halo/SNAP tagged cells.
    i. Add 1 μM ligand (Janelia Fluor JFX650 HaloTag or SNAP-Cell 647-SiR) in a total volume of 120 μL of pre-warmed growth medium in the 14 mm micro-well at the center of the dish, incubate for 1 h. at 37 °C with 5% CO_2_.
    ii. Wash 3X with 2 mL of pre-warmed growth medium.
    iii. Incubate for 60 min. at 37 °C with 5% CO_2_ to allow any unbound dye to leach out of the cells into the medium.
      ***Note:*** SNAP and Halo substrates are available in a palette of colors from New England Biolabs (SNAP) and Promega (Halo). For alternative SNAP and Halo labeling strategies see.^1^^,^^2^^,^^4^^,^^5^
      ***Note:*** mStayGold is a fluorescent protein and does not require staining.
    iv. Shortly before imaging, wash cells with 2 mL PBS and add 2 mL pre-warmed live-cell imaging solution.
    v. Image cells on a light microscope using settings to detect fluorophores in the far-red range (for Janelia Fluor JFX650 HaloTag or SNAP-Cell 647-SiR ligands; 640-650 nm laser) or green range (for mStayGold; 488 nm laser) to assess the localization of the protein of interest. Troubleshooting 5, 6, 7, and 8.
      ***Note:*** We recommend using a light microscope with laser sources as high laser intensities may be required to visualize low abundance proteins.
      ***Note:*** Examples of confocal images of different KI cell lines expressing endogenously tagged proteins localizing to different compartments are shown in Figure 17.
      ***Alternatives*:** A primary antibody against the inserted Tag (for recommended anti-V5/ALFA/HA/Halo/SNAP antibodies see key resources table) in combination with a secondary antibody against the primary antibody carrying a fluorophore can be used for immunofluorescence.

### Further selection of edited cells

**Timing: 1 week–1 month**

If the editing efficiency is insufficient or homozygous clones are required, cells edited with SNAP, Halo or mStayGold can be further enriched through fluorescence activated cell sorting (FACS) (Step 32). Following FACS enrichment, cells can be validated via Western blotting (see Step 28), in-gel fluorescent detection of the tagged proteins (Step 29) and imaging (Step 31). Clones derived from single cells can be genotyped to assess zygosity (see Step 33).

***Note:*** We recommend validating genotyped clones via Western blotting using an antibody specific for the protein of interest as genotyping primers may not be able to bind to genomic sequences altered by NHEJ.

**Figure 18:**
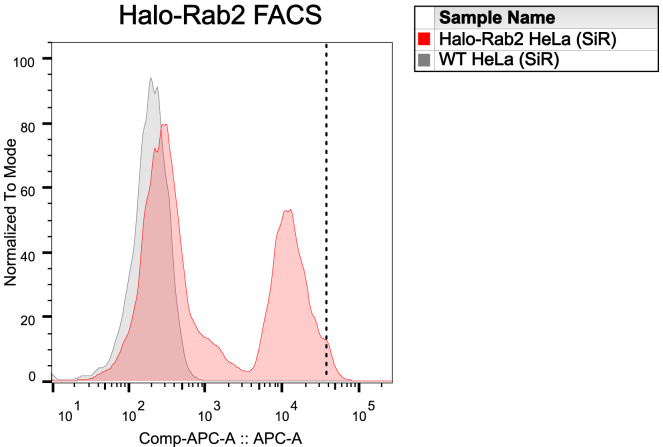
Example histogram FACS blots of Halo-Rab2^EN^ HeLa cells Halo-Rab2^EN^ (red curve) and WT (negative control, gray curve) HeLa cells were stained with SiR-Halo and sorted. Gating was set as shown by the dotted line to sort for the 1% brightest cells. EN=endogenous.
33. Genotyping of clonal cell lines derived from single cells.
  a. Amplify the target locus as described in Step 30.
  b. Load 5 μL of the PCR product on an agarose gel and use WT cells as a negative control.
  c. Assess the DNA patterns on the gel.

***Note:*** Amplification of the WT locus should yield a low molecular weight band (see WT lane in Figure 19 of an example gel picture). Amplification of the edited locus should yield a higher molecular weight band because of the insertion of a large fragment (*e.g.,* Halo) via editing. Heterozygous clones will show 2 bands (one for the edited allele(s) and one for the WT allele(s), lane C7 and C11) while homozygous clones will show a single higher molecular weight band as all alleles are edited (lane C6 and C9).

32. Further enrichment for edited cell with FACS.
  a. Seed cells for FACS.
    i. Seed edited cells in a T75 flask; a confluent flask is required on the day of sorting.
  b. Cell preparation for FACS.
    i. On the day of sorting, label gene-edited cells with 0.5 μM SNAP/Halo ligands in growth medium as described in Step 31b.
      ***Note:*** Typically, we stain a confluent T75 flask in 5 mL of growth medium to reduce dye usage.
    ii. Sort labeled cells via FACS, gating on the 0.5–1% brightest cells to select for homozygous edited cells.
      ***Note:*** An exemplary FACS plot is shown in Figure 18.
      ***Note:*** For single-cell sorting, single cells can be collected in each well of a 96-well plate and grown to confluency.

**CRITICAL:** When working with clones derived from single cells, we recommend using several clones for downstream experiments to ensure any observed effects are not due to clonal selection.

**Figure 19:**
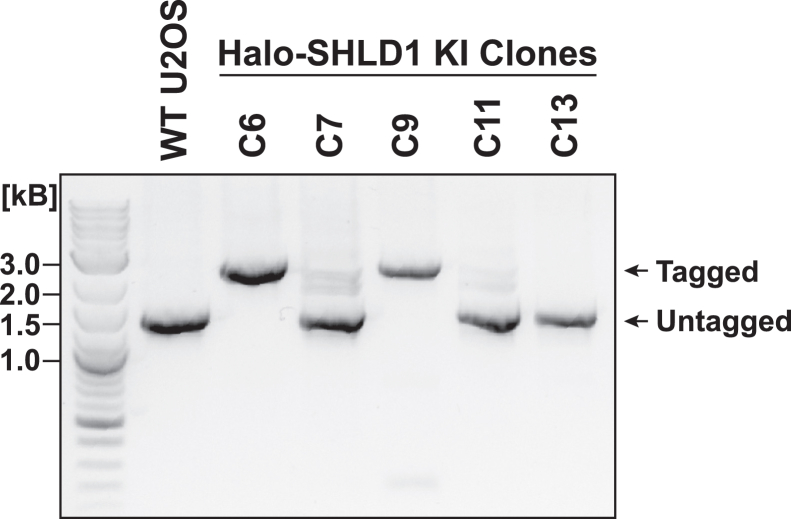
Example image of a DNA gel used for genotyping of 3xFLAG-Halo edited Shieldin Complex Subunit 1 (Halo-SHLD1) U20S cell clones PCR with primers flanking the edited locus yields a single lower molecular weight band in wild-type (WT U2OS) unedited cells, corresponding to the unedited locus. Clone 7 and 11 (C7, C11) are heterozygous clones, displaying both the lower molecular weight band (untagged/unedited locus) and a higher molecular weight band (tagged/edited locus). Clone 6 and 9 (C6, C9) are homozygous clones, showing only the higher molecular weight band corresponding to the edited locus. Clone 13 is unedited, displaying only a low molecular weight band corresponding to the WT allele.

### Functional verification of the endogenously tagged protein

**Timing: variable**

34. Ensure the Tag does not disrupt functionality of the protein by using homozygous clones where both alleles have been edited and performing a suited experiment.

***Note:*** The required experiments depend on the biological role of the protein. For example, trafficking assays are suitable to test the functionality of proteins involved in membrane trafficking.

***Alternatives*:** A haploid cell line like HAP1 can be used, where a single editing event yields 100% tagged products.^1^^,^^34^

## Expected outcomes

C-terminal tagging with FAB-CRISPR should yield KI efficiencies (percentage of cells with at least 1 edited allele in a population of cells) close to 100% in actively proliferating and easy-to-transfect cell lines. Some variability is expected depending on the chosen targeting gRNA and intrinsic cell parameters (*e.g.,* chromatin accessibility, cell type, etc.). Under optimal conditions, for N-terminal tagging the final KI efficiency will depend on the transfection efficiency of the EGFP-Cre recombinase plasmid used to excise the antibiotic resistance cassette as Cre recombinase is highly efficient. However, efficiencies as high as 80% can be achieved when both transfection steps are optimally performed. Nevertheless, editing of mammalian cells will yield mostly monoallelic modifications as HDR is a rare event, complicating editing in cell lines with higher ploidity. We recommend reading the troubleshooting section for tips on how to improve KI efficiencies to ensure optimal experimental conditions. Finally, this method could also be used for inserting tags in the middle of a protein sequence. In this case, an excisable LoxP-flanked resistance cassette may be placed within an intron neighboring the insertion site.

## Limitations

Endogenous expression levels are moderate when compared to expression levels achieved via plasmid overexpression. This needs to be accounted for when performing downstream experiments (*e.g.,* for mass spectrometry experiments one may have to increase the amount of cell material used). Various tools are available for checking protein and transcript abundance (but bear in mind that mRNA levels do not always correlate with protein levels!), *e.g.*, http://mapofthecell.biochem.mpg.de/index.html^35^ and https://www.proteinatlas.org/^36^

Tagging at the N-terminus using the excisable resistance cassette strategy leads to a transient KO of the protein of interest as the locus is disrupted and proteins are not expressed. Upon excision of the resistance cassette using EGFP-Cre, expression of the target protein should be restored (validate your cell lines using the suggested methods). One of the two LoxP sites remains at the insertion site but in our experience, this had no deleterious effects on protein expression. For essential genes, this strategy will likely only yield heterozygous KIs, as homozygous KIs will not be viable.

Moreover, when trying to edit essential genes, tagging at the C-terminus is less likely to disrupt protein expression because the protein is likely expressed during the antibiotic selection. Tagging the N-terminus of essential genes can cause a loss of protein functionality. If it must be tagged N-terminally, we advise direct N-terminal insertion of the Tag devoid of the LoxP-AB^R^-LoxP cassette. This strategy overcomes the temporary non-functionality of the expressed protein. A caveat of direct insertion is that the lack of the antibiotic selection marker may reduce the knock-in efficiency. In this case, FACS (Step 32) can then be used to enrich for the gene-edited cells. In the design provided in this protocol, the LoxP-AB^R^-LoxP cassette is inserted upstream of the endogenous ATG start codon. To avoid changing any bases prior to the start codon, since they may contain sequences that are important for transcription and translational efficiency, we have successfully inserted the LoxP-flanked cassette downstream of the endogenous start codon.^31^ Last, when using a non-fluorescent Tag, such as Turbo-ID, FACS is not possible and single clone selection will have to be carried out manually via serial dilution.

## Troubleshooting

### Problem 1

Unexpected fragment pattern observed in HDR donor plasmid digestion (Step 11).

### Potential solution

Ensure the LHA, RHA, AB^R^, Tags, and plasmid backbone sequences do not include restriction sites needed for cloning of the final HDR donor plasmid. This can be done by performing an *in silico* digest on the assembled target sequence with SnapGene or a molecular biology tool of choice. If a restriction site is present within an intron or coding sequence, the sequence in the HDR donor plasmid can be mutagenized. Within an intron, beware of changing bases at the exon-intron junctions. Within the coding sequence, insert a silent mutation to not change the aminoacidic sequence. If this is not feasible, desired restriction sites can be introduced by PCR amplification of the FAB-CRISPR insert (use FAB-CRISPR plasmids as a template) with primers that encode the restriction sites, as well as a 20 bp overlap with the beginning (sense primer) or end (anti-sense primer) of the FAB-CRISPR insert.

### Problem 2

No cells survive the antibiotic treatment (Step 26).

### Potential solution

• Troubleshoot the transfection method with a fluorescent marker (*e.g.*, EGFP-tagged overexpression protein).
• Check the quality of the used DNA Mini/Maxipreps.
• When tagging at the N-terminus using the excisable resistance cassette strategy, the tagged protein won’t be expressed until the resistance cassette is properly excised. Thus, the lack of surviving cells may indicate that the targeted gene is essential for cell viability.
• Check the cutting efficiency of your gRNAs with a surveyor assay.^30^

### Problem 3

No or only a few cells are killed by the antibiotic treatment (Step 26).

### Potential solution

If the cells grew too confluent during the antibiotic treatment, the selection efficiency may be reduced (we have particularly observed this when using G418). We have observed that lengthening the antibiotic treatment leads to the appearance of cells that do not expressed the tagged protein but are resistant to the antibiotic. These cells could have randomly integrated the resistance cassette and/or have sustained expression of the plasmid DNA. In this case, we recommend repeating the transfection (Step 25) onward and ensure that the cells are less than 50% confluent when starting the antibiotic selection. Further, ensure that only a small percentage of edited cells survive the antibiotic treatment. Trypsinization of the cells prior antibiotic addition improves selection. If necessary, increase the antibiotic concentration (for G418 up to 3 mg/mL) during the first few days of selection.

### Problem 4

Replacement of endogenous with an exogenous polyA sequence (C-terminal tagging) may alter protein expressions when the amount of transcript is tightly regulated at the mRNA level (“Designing a homology-directed repair donor (HDR) plasmid for N- or C-terminal tagging”). This can result in altered expression levels (protein levels significantly higher or lower than the endogenous expression levels detected in WT cells) of C-terminally tagged proteins (Step 28).

### Potential solution

Construct your HDR donor plasmid to include an excisable resistance cassette (LoxP-AB^R^-LoxP) downstream of the STOP codon of the Tag. This allows the resistance cassette to be removed post-selection.

### Problem 5

No endogenously tagged protein is detected via Western blotting, in-gel fluorescence and/or via fluorescence microscopy (Step 28, 29, 31).

### Potential solution

• Adding a high-affinity epitope Tag along with Halo/SNAP/mStayGold (ALFA/V5/HA) may simplify detection and allow signal amplification via immunofluorescence and/or Western blotting.
• Use high sensitivity Western blot detection reagents.
• Optimize excitation and detection settings on the fluorescence microscope.

### Problem 6

Your Halo/SNAP-tagged protein is expressed at very low endogenous levels and the background of cell-permeable Halo/SNAP substrates (Step 31) masks the real signal (*e.g.,* bright structures due to endocytosed dye).

### Potential solution

• Test the amount of dye background by staining unedited WT cells.
• Leave excess dye to wash out for longer times (> 1 h.) or optimize dye concentration.
• Test the localization of the protein of interest tagged with mStayGold.

### Problem 7

Tagging interferes with protein localization and function (Step 31).

### Potential solution

• Increase the length of the linker between the target protein and the Tag for example by using a localization and affinity purification (LAP) Tag linker.^1^^,^^37^
• If working with single cell clones, we recommend performing the same experiments using additional clones to rule out clonal differences.

### Problem 8

The CRISPR KI efficiency is too low (Step 28–31).

### Potential solution

Low KI efficiency may be due to different factors.

• Antibiotic selection has not worked efficiently (see problem 3).
• The gRNA is not cutting efficiently. For this, test other gRNAs and test their efficiency of gRNA targeting with the surveyor assay^30^
• For N-terminally tagged proteins, inefficient excision of the LoxP-AB^R^-LoxP cassette by the EGFP-Cre recombinase lowers the percentage of cells expressing the tagged proteins. In this case, we recommend troubleshooting the transfection of the EGFP-Cre recombinase by monitoring nuclear EGFP expression.

## Resource availability

### Lead contact

Francesca Bottanelli: francesca.bottanelli@fu-berlin.de.

### Technical contact

petiaa97@zedat.fu-berlin.de.

### Materials availability

Plasmids generated in this study have been deposited to Addgene and all catalog numbers are indicated in the resources table.

### Data and code availability

This study did not generate/analyze any datasets/code.

## Acknowledgments

We acknowledge all the members of the Bottanelli and Schmidt laboratories for supporting the development of the tools explained in this protocol. We offer special thanks to Pia Hagenbach for providing the FACS blots. P.A. is supported by the Deutsche Forschungsgemeinschaft (DFG)—project number 278001972—TRR 186.

## Author contributions

P.A., E.F., J.H., and C.B. conducted the experiments and analyzed the data. J.S. and F.B. supervised the project. P.A., E.F., J.H., C.B., J.S., and F.B. wrote the original draft and revised and edited the manuscript.

## Declaration of interests

The authors declare no competing interests.
