# Supplementary material for: Protocol for fast antibiotic resistance-based gene editing of mammalian cells with CRISPR-Cas9
Source: STAR Protoc. 2025 Aug 4;6(3):103949. doi: 10.1016/j.xpro.2025.103949 (PMC12345277; doi:10.1016/j.xpro.2025.103949)
Supplement: Document S1. Figures S1–S3 [file mmc1.pdf]

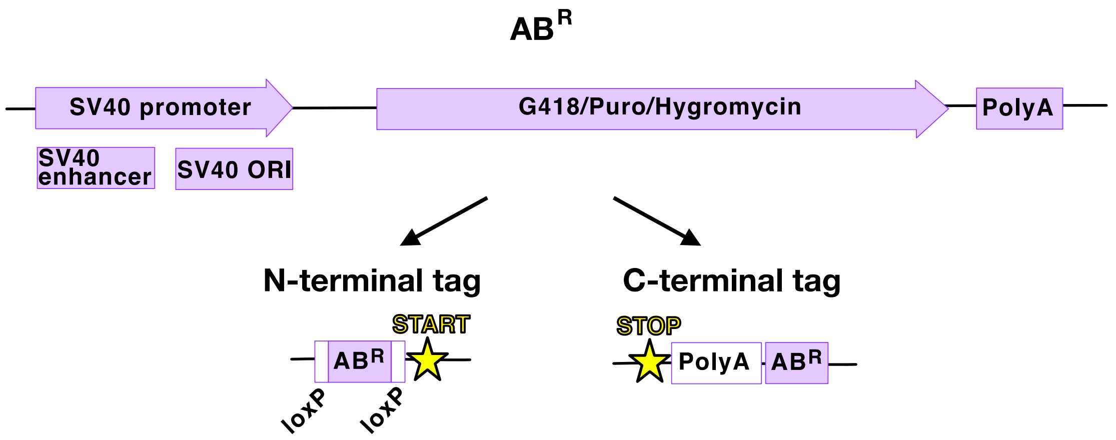

Figure S1: Components of the antibiotic resistance cassettes (AB^R), related to Preparation Step 14 and Steps 9 and 15. AB^R consists of a SV40 promoter, a gene encoding for a protein conferring the antibiotic resistance (G418/Puromycin/Hygromycin) and a Polyadenylation signal (polyA) - SV40 polyA for the Puromycin/Hygromycin plasmid and HSV TK polyA for the G418 plasmids. Depending on the tagging strategy, the AB^R is either flanked by LoxP sites and can be excised using Cre recombinase (N-terminal fusion) or is present downstream of an exogenous SV40 polyA signal (C-terminal fusion).

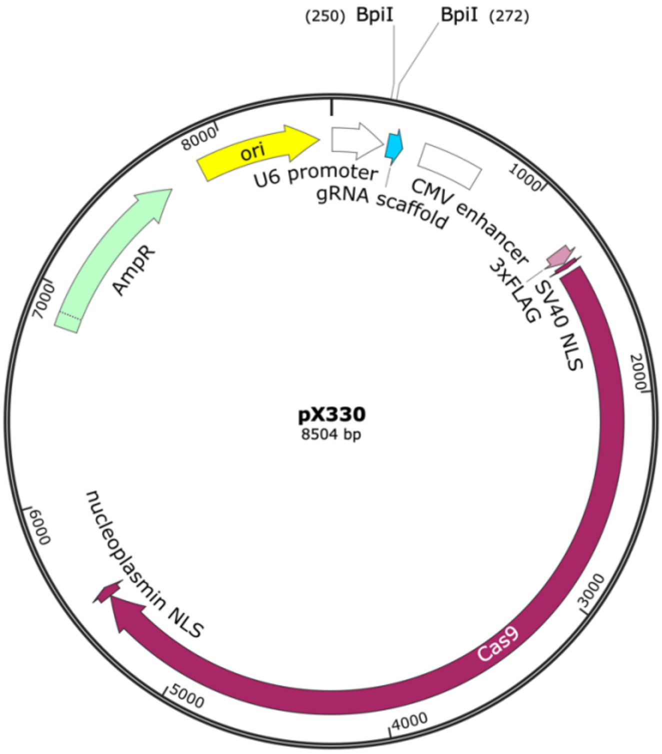

Figure S2: pX330 plasmid map (Addgene #42230), related to Preparation Step 12 and Step 2. The desired guide (gRNA scaffold, blue) can be inserted in between the BpiI sites downstream of the U6 promoter. Map is visualised using the SnapGene® software.

A

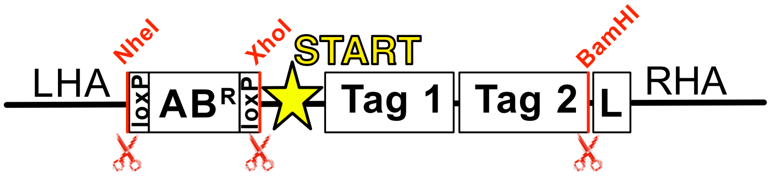

Addgene ID	Restriction site	Insert	Restriction site	Size
229676	NheI	LoxP-G418-LoxP	XhoI	1516 bp
229677	NheI	LoxP-Puromycin-LoxP	XhoI	1487 bp
	XhoI	3xV5-SNAP	BamHI	716 bp
229678	NheI	LoxP-Hygromycin-LoxP	XhoI	1696 bp
	XhoI	HA-mStayGold	BamHI	711 bp

Rab11 Backbone size: 3671 bp

B

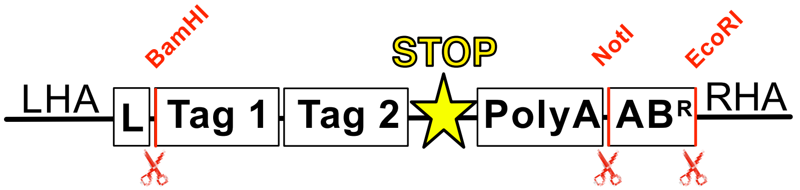

Addgene ID	Restriction site	Insert	Restriction site	Size
227734	BamHI	TurboID-V5-PolyA	NotI	1273 bp
	NotI	G418	EcoRI	1449 bp
229681	BamHI	Halo-ALFA-PolyA	NotI	1207 bp
	NotI	G418	EcoRI	1428 bp
229680	BamHI	SNAP-V5-PolyA	NotI	856 bp
	NotI	Puromycin	EcoRI	1398 bp
229679	BamHI	mStayGold-PolyA	NotI	949 bp
	NotI	Hygromycin	EcoRI	1629 bp

AP1μA Backbone size: 4379 bp

C

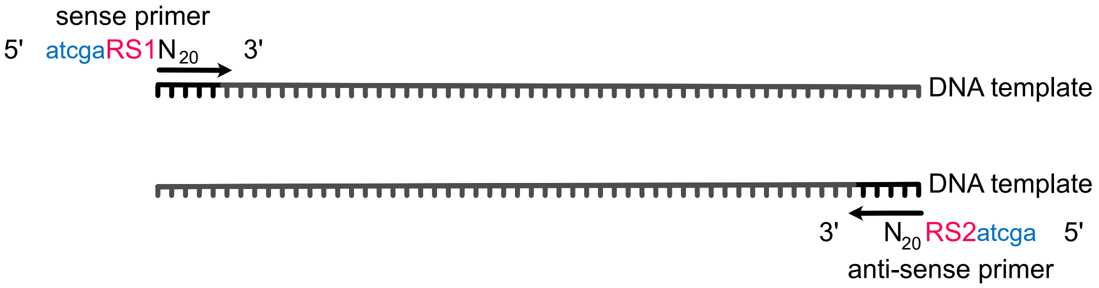

Figure S3: Overview of fragments for the generation of customized HDR plasmids, related to Steps 17 and 18. A The shown N-terminal Rab11 HDR donor plasmids contain a XhoI site which can be used to exchange the antibiotic resistance cassette (AB^R) or Tags. Due to additional XhoI sites in the LHA and RHA of the Rab11 HDR plasmid, cutting with XhoI will generate multiple fragments: 565 bp, 2773 bp, 333 bp. To be noted: the Halo Tag (Addgene ID 229676) sequence has an internal XhoI restriction site. **B** All C-terminal AP1μA HDR donor plasmids contain a NotI site which can be used to exchange either

the AB^R or Tags. **C** For generating custom inserts, the FAB-CRISPR HR donor plasmids or another plasmid encoding for the desired insert can be used as a DNA template and amplified via PCR using a sense and anti-sense primer. For this, primers need to contain 20 nucleotides (N_{20}) that are complementary to the DNA template (black), a unique restriction site (RS1 or RS2) and random base pairs (blue).
